# Isocitrate dehydrogenase 1 mutation drives leukemogenesis by *PDGFRA* activation due to insulator disruption in acute myeloid leukemia (AML)

**DOI:** 10.1038/s41375-022-01751-6

**Published:** 2022-11-21

**Authors:** Sophie Steinhäuser, Patricia Silva, Lennart Lenk, Thomas Beder, Alina Hartmann, Sonja Hänzelmann, Lars Fransecky, Martin Neumann, Lorenz Bastian, Simone Lipinski, Kathrin Richter, Miriam Bultmann, Emely Hübner, Shuli Xia, Christoph Röllig, Fotini Vogiatzi, Denis Martin Schewe, Veronica Yumiceba, Kristin Schultz, Malte Spielmann, Claudia Dorothea Baldus

**Affiliations:** 1 Department of Inner Medicine II (Hematology/Oncology), University Hospital Schleswig-Holstein, Kiel, Germany; 2 Department of Hematology and Oncology, Charité University Hospital, Berlin, Germany; 3 Department of Pediatrics I, ALL-BFM Study Group, University Hospital Schleswig-Holstein, Kiel, Germany; 4 University Cancer Center Schleswig-Holstein (UCCSH), University Hospital Schleswig-Holstein, Kiel, Germany; 5 Kennedy Krieger Institute, Baltimore, MD USA; 6 School of Medicine, Department of Neurology, Johns Hopkins University, Baltimore, MD USA; 7 Department of Internal Medicine I, University Hospital Carl-Gustav-Carus, Dresden, Germany; 8 Children´s Hospital, University Hospital Magdeburg, Magdeburg, Germany; 9 Institute for Human Genetics, University Hospital Schleswig-Holstein, Lübeck, Germany

**Keywords:** Translational research, Acute myeloid leukaemia, Cancer genomics

## Abstract

Acute myeloid leukemia (AML) is characterized by complex molecular alterations and driver mutations. Elderly patients show increased frequencies of *IDH* mutations with high chemoresistance and relapse rates despite recent therapeutic advances. Besides being associated with global promoter hypermethylation, *IDH1* mutation facilitated changes in 3D DNA-conformation by CTCF-anchor methylation and upregulated oncogene expression in glioma, correlating with poor prognosis. Here, we investigated the role of *IDH1* p.R132H mutation in altering 3D DNA-architecture and subsequent oncogene activation in AML. Using public RNA-Seq data, we identified upregulation of tyrosine kinase *PDGFRA* in *IDH1*-mutant patients, correlating with poor prognosis. DNA methylation analysis identified CpG hypermethylation within a CTCF-anchor upstream of *PDGFRA* in *IDH1*-mutant patients. Increased *PDGFRA* expression, *PDGFRA*-CTCF methylation and decreased CTCF binding were confirmed in AML CRISPR cells with heterozygous *IDH1* p.R132H mutation and upon exogenous 2-HG treatment. *IDH1*-mutant cells showed higher sensitivity to tyrosine kinase inhibitor dasatinib, which was supported by reduced blast count in a patient with refractory *IDH1*-mutant AML after dasatinib treatment. Our data illustrate that *IDH1* p.R132H mutation leads to CTCF hypermethylation, disrupting DNA-looping and insulation of *PDGFRA*, resulting in *PDGFRA* upregulation in *IDH1*-mutant AML. Treatment with dasatinib may offer a novel treatment strategy for *IDH1*-mutant AML.

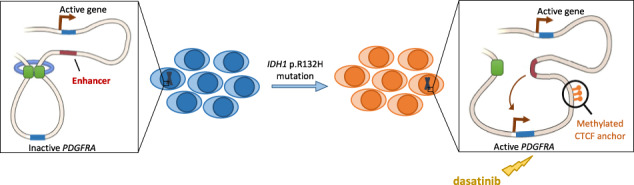

## Introduction

Acute myeloid leukemia (AML) describes a myeloid disorder that is characterized by oncogenic transformation and uncontrolled clonal expansion of poorly differentiated cells of myeloid origin [1]. Among the adult population, AML is one of the most common types of leukemia [2]. On the molecular level, AML represents a largely heterogeneous disease, characterized by complex patterns of molecular alterations such as gene fusions or rearrangements together with somatically acquired driver mutations [3]. The advancing knowledge of molecular pathogenesis in AML led to the proposal of molecular classifications currently dividing AML into 11 subgroups by cytogenetic aberrations and different prognostic markers [4]. Frequently mutated genes belong to numerous functional categories including signaling genes (*FLT3*, *KRAS*, *NRAS*), nucleophosmin (*NPM1*), myeloid transcription factors (*RUNX1*, *CEBPA*), chromatin regulators (*ASXL1*) or DNA methylation-associated genes and epigenetic regulators (*DNMT3A*, *TET2*, *IDH1/2*) [3].

In younger adults, *IDH1* mutations occur in 6-10% of AML cases, whereas elderly AML patients show a significantly higher *IDH1* mutation frequency of ~17% accompanied by global DNA hypermethylation [5]. *IDH1* and *IDH2* catalyze the conversion of isocitrate to α-ketoglutarate (α-KG) in the cytoplasm (*IDH1*) or the mitochondria (*IDH2*) as part of the citrate cycle. α-KG further activates enzymes of the ten-eleven translocation (TET) family in the nucleus that are essential mediators of DNA demethylation and subsequent transcriptional activation of target genes [1, 6–8]. The most common mutation reported for *IDH1* is p.R132H, resulting from a G to A base shift that leads to an amino acid shift from arginine at position 132 to histidine and a subsequent conformation change in the IDH1 protein [6]. As a result, an alternative oncometabolite 2-hydroxygluterate (2-HG) is generated, which competes with α-KG for binding at the TET enzymes while it lacks the ability to activate them. Subsequently, TET enzyme activity is inhibited, resulting in inhibition of demethylation and therefore a hypermethylation phenotype [1, 5, 9]. Studies have shown that such hypermethylation is often found at promoters of genes involved in cell differentiation, connecting the occurrence of *IDH* mutations to the inhibition of myeloid differentiation [7]. The recent approval of the therapeutic drugs ivosidenib (*IDH1*) and enasidenib (*IDH2*) by the Food and Drug Administration (FDA) has been an important advance in the treatment of patients with AML patients containing *IDH* mutations [10]. However, resistance and relapse still remain a problem to be addressed [11].

In addition to a differentiation block mediated by hypermethylated gene promotors, recent research suggests an alternative mechanism of action through which *IDH1* mutations may contribute to tumorigenesis. In glioma, the presence of *IDH1* mutation was associated with CpG hypermethylation of binding sites for the insulator protein CTCF (CCCTC-binding factor), disabling CTCF binding and correct establishment of DNA loops. As a result, 3D conformational changes of the DNA-architecture affect long-range interactions and subsequently deregulate gene expression [12]. Dysregulation of long-range DNA interactions was recently shown to be involved in leukemogenesis [13, 14]. However, the activation of defined oncogenes by hypermethylation of CTCF sites in the context of *IDH1*-mutated AML has not been explored. A better understanding of these mechanisms will potentially unravel novel therapeutic targets in the treatment of AML with *IDH1* mutation, contributing to overcoming resistance and improving patient outcome.

The aim of this study was to investigate the impact of the *IDH1* p.R132H mutation by CTCF site hypermethylation, disruption of 3D DNA conformation and subsequent gene expression changes in AML. Differential gene expression was analyzed in public AML RNA-Seq [15, 16] and microarray data [17] in order to identify deregulated cancer-associated genes in patients with *IDH1* mutation. Differential expression results were then integrated with previously generated DNA methylation data [5] and public ChIP-Seq as well as CHIA-PET data in order to correlate upregulated oncogenes with CTCF hypermethylation in the context of altered DNA loop formation. The results were then used for exploration of novel therapeutic options for specific treatment of patients with *IDH1*-mut AML.

## Methods

### Cell culture

KG1a myeloid leukemia cells contain a FGFR1OP2-FGFR1 gene fusion but lack other known driver mutations or genetic alterations and were therefore selected as cellular model. Cells were obtained from the German Collection of Microorganisms and Cell Cultures (DSMZ, Braunschweig, Germany), maintained at 0.2–2.0 million cells/ml in RPMI-1640 culture medium (Gibco, Billings, MT, United States) with 20% fetal bovine serum and checked regularly for mycoplasma.

### CRISPR base editing and CRISPR knockout

To generate an *IDH1* point mutation in KG1a cells, we applied CRISPR base editing [18]. For CRISPR knockout of *PDGFRA* in *IDH1*-mut cell clones and knockout of the *PDGFRA*-CTCF-anchor site in *IDH1*-wt clones, synthetic sgRNAs were purchased from Synthego (CA, USA) and electroporated together with Cas9 RNP. A detailed description can be found in the Supplementary Methods section. Sanger sequencing primers and sgRNAs are listed in Supplementary Table 1.

### RNA, cDNA, RT-qPCR

A detailed description can be found in the Supplementary Methods section. Relative gene expression was calculated using the ΔΔCt method according to the MIQE guidelines [19].

### Differential gene expression and survival analysis

Differential gene expression was analyzed in R v4.0.3 with VST values generated from RNA-Seq raw counts using DESeq2 [20]. A detailed description of all used datasets for differential expression and survival analysis can be found in Supplementary Methods section.

### DNA methylation and 3D DNA-conformation data

DNA methylation of patients with *IDH1*-mut and *IDH1*-wt AML from the Study Alliance Leukemia (SAL) elderly AML cohort (*n* = 79) was assessed using the Infinium® HumanMethylation450 BeadChip platform and combined with the TCGA AML cohort (*n* = 194) for comprehensive methylation analysis as described previously [5]. Methylation data are accessible through NCBI’s Gene Expression Omnibus GSE86409. A detailed description can be found in Supplementary Methods section.

### Methylation-sensitive restriction digest

A detailed description can be found in the Supplementary Methods section.

### Assessment of CTCF binding using ChIP-qPCR

A detailed description can be found in the Supplementary Methods section.

### In vitro drug sensitivity screenings, 2-HG detection and treatment

A detailed description can be found in the Supplementary Methods section.

### In vivo assessment of dasatinib sensitivity

In vivo experiments were performed using busulfan-pretreated NSG mice [21–26] based on the ethical approval for animal studies TVA-V 242—42134/2021 (53-7/21) from the Ministerium für Energiewende, Landwirtschaft, Umwelt, Natur und Digitalisierung (MELUND), Schleswig-Holstein, Germany. A detailed description can be found in the Supplementary Methods.

### Single-case AML patient data

Peripheral blood from a 54-years old patient with AML enrolled in the Study Alliance Leukemia (SAL) registry was collected for analysis of neutrophil and leukemic blast counts after informed written consent in accordance with the declaration of Helsinki approved by the local institutional review boards. The patient acquired an *IDH1* mutation after failure of 4 lines of therapy, including allogeneic hematopoietic stem cell transplantation. *IDH1*-positive AML was treated with venetoclax/azacytidine, DLI and cytarabine (araC)/mitoxantrone but resulted in refractory disease. Therefore, therapy with dasatinib was initiated as compassionate use while approval of ivosidenib treatment as compassionate use was awaited. An executive summary of the patient history is provided in Supplementary Table 2.

### Statistical analysis

All experiments were performed at minimum in triplicate. Graphs were generated in Microsoft Excel 2016. Error bars represent the standard deviation of the mean. Statistical analysis was performed in R v4.0.3. (R Development Core team, 2013). Data were checked for normal distribution and two-sided Students t-test or ANOVA was performed when normally distributed whereas Wilcoxon or Mann–Whitney U test was used when not normally distributed. The significance level was 0.05.

## Results

### Upregulation of receptor tyrosine kinase *PDGFRA* in *IDH1*-mut AML

**Fig. 1:**
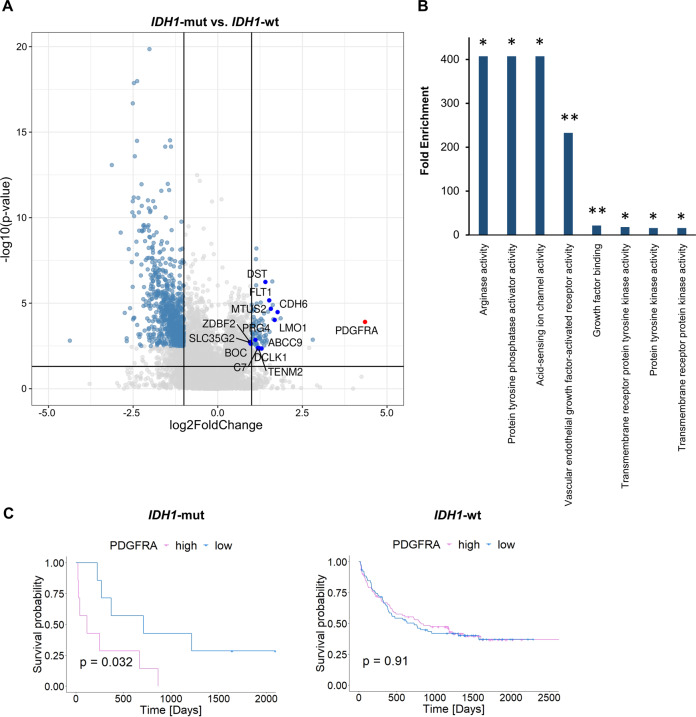
Oncogene expression in *IDH1*-mut AML. **A** Differential gene expression in *IDH1*-mut vs. *IDH1*-wt AML (data: BeatAML [15]. *IDH1*-mut patients: *n* = 28. *IDH1*-wt patients: *n* = 153). Blue dots = Differentially expressed genes between *IDH1*-wt and *IDH1*-mut AML with cut-off of p.adjusted < 0.05 and log2foldchange > 1. Labeled genes = upregulated cancer-associated genes according to the COSMIC Cancer Gene Census (CGC), the OncoKB and the Network of Cancer Genes databases (NCG 7.0). **B** Enrichment of cellular interaction-related functional gene groups in *IDH1*-mut upregulated genes. Gene Ontology enrichment analysis (ShinyGO v0.75) was performed using GO Molecular Function with an FDR cut-off of 0.05. Fold enrichment of GO groups enriched among *IDH1*-mut upregulated genes is plotted with **FDR < 0.01 and *FDR < 0.05. **C** Overall survival of *IDH1*-mut AML patients with high/low *PDGFRA* expression (*n* = 8 vs. *n* = 8) versus *IDH1*-wt patients with high/low *PDGFRA* (*n* = 68 vs. *n* = 68). *PDGFRA* high: samples with *PDGFRA* expression above median. *PDGFRA* low: samples below median. Method: Kaplan-Maier, significance cut-off *p* < 0.05. Clinical data: Bamopoulos et al. [16].

In order to identify upregulated genes in *IDH1*-mut AML that contribute to *IDH1*-driven leukemogenesis, we analyzed differential gene expression between patients with *IDH1*-wt (*n* = 153) and *IDH1*-mut (*n* = 28) AML with hotspot R132 mutation within the BeatAML RNA-Seq dataset [15]. Differential expression analysis identified a total of 132 upregulated genes (log2FoldChange > 1, *p*-adjusted < 0.05) in patients with *IDH1*-mut compared to *IDH1*-wt AML (Supplementary Table 4). Screening of these upregulated genes for classified cancer-associated genes according to the COSMIC Cancer Gene Census (CGC), the OncoKB and the Network of Cancer Genes databases (NCG 7.0) revealed the receptor tyrosine kinase *PDGFRA* (platelet-derived growth factor alpha) as the most upregulated oncogene in *IDH1*-mut AML (Fig. 1A). In contrast, *PDGFRA* expression was not significantly upregulated in *IDH2*-mut AML (Supplementary Fig. S1A). Gene set enrichment analysis for GO Molecular Function among upregulated genes in patients with *IDH1*-mut AML revealed enrichment of receptor tyrosine kinase (RTK) activity as well as growth factor binding (Fig. 1B), while no functional categories were significantly enriched among upregulated genes in *IDH2*-mut AML. Significant upregulation of *PDGFRA* in *IDH1*-mut AML was further confirmed using an independent AML gene expression microarray dataset published by Verhaak et al. (Supplementary Fig. S1B) [17]. In order to determine clinical relevance of upregulated *PDGFRA* expression, we compared overall survival of patients with *IDH1*-mut AML with high (above median) or low (below median) *PDGFRA* expression (*n* = 8 versus *n* = 8) as well as *IDH1*-wt patient with high or low *PDGFRA* (*n* = 68 versus *n* = 68) using published clinical data by Bamopoulos et al. [16]. Survival analysis revealed inferior survival of patients with high *PDGFRA* expression among *IDH1*-mut but not *IDH1*-wt groups (Fig. 1C, *p* = 0.032 vs. *p* = 0.91). In contrast, no survival difference with respect to *PDGFRA* expression was found in *IDH2*-mut versus *IDH2*-wt groups (Supplementary Fig. S2A). This was further confirmed using the BeatAML clinical data [15] (Supplementary Fig. S2B).

### Increased CpG hypermethylation within *PDGFRA*-CTCF anchor in *IDH1*-mut AML

**Fig. 2:**
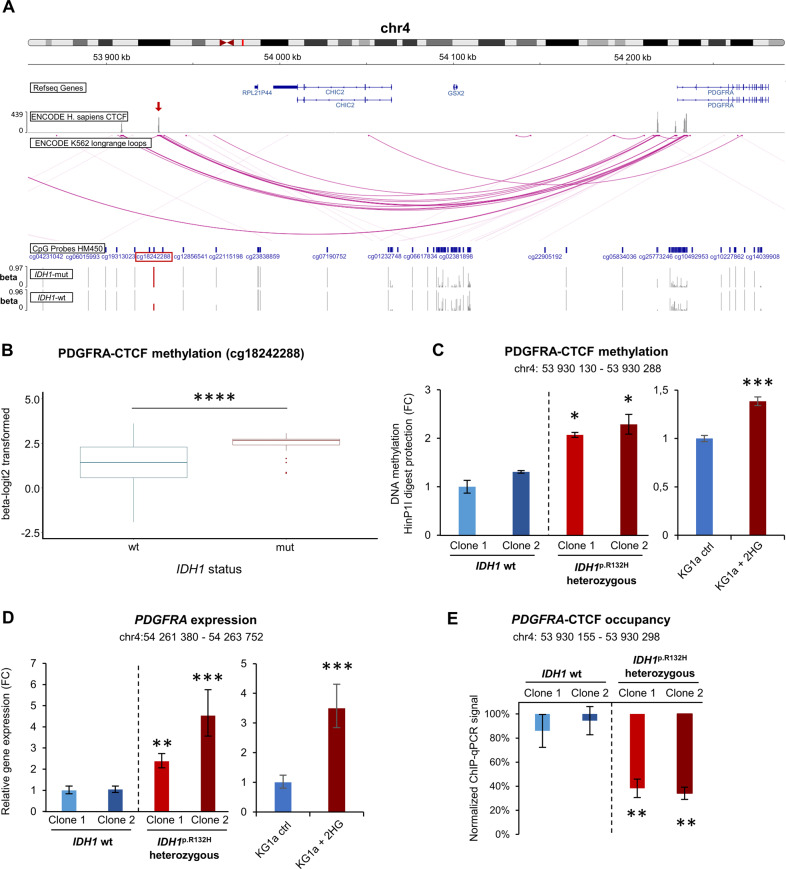
Hypermethylation and decreased CTCF binding at the CTCF loop anchor upstream of *PDGFRA* locus in *IDH1*-mut AML. **A** Top: Visualization of CTCF-anchor site and long-range interaction loops using IGV Genome Browser with ENCODE ChIP-Seq annotation tracks for human CTCF and ENCODE CHIA-PET annotations of CTCF long-range interactions for K562 leukemia cells (hg38) [46]. Methylation tracks (beta-values) of *IDH1*-wt and *IDH1*-mut AML patient samples [5] for assessment of CpG methylation within CTCF-anchor site upstream of *PDGFRA* gene locus (red arrow) using Illumina HM450 data from *IDH1*-wt (*n* = 214) and *IDH1*-mut (*n* = 33) AML patients [5]. CpG methylation peaks at mentioned CTCF-anchor site (cg18242288) were highlighted in red. **B** Assessment of CpG methylation within CTCF-anchor site upstream of *PDGFRA* gene locus using Illumina HM450 data from *IDH1*-wt (*n* = 214) and *IDH1*-mut (*n* = 33) AML patients [5]. Statistical analysis of beta-logit2 transformed values (M-values) for Illumina HM450 CpG probe cg18242288 using Wilcoxon test with Benjamini Hochberg FDR correction. ****p < 0.0001. **C** In vitro confirmation of *PDGFRA*-CTCF hypermethylation based on protection from methylation-sensitive HinP1I digestion of *IDH1*-wt/-mut KG1a cell clones (2 clones, 3 replicates) and 2-HG treated naïve KG1a cells (3 replicates). Data (mean ± SD) display fold change (FC) in relative expression, Students T-test, *p < 0.05; **p < 0.01; ***p < 0.001. **D** RT-qPCR confirmation of *PDGFRA* upregulation in *IDH1*-mut KG1a cell clones (2 clones, 3 replicates) and 2-HG treated naïve KG1a cells (3 replicates). Data display fold change (FC) in relative expression, Students T-test, *p < 0.05; **p < 0.01; ***p < 0.001. **E** Decreased CTCF occupancy of *PDGFRA*-CTCF-anchor region in *IDH1*-mut KG1a cell clones (2 clones, 3 replicates). Data represent mean ± SD. Students T-test, **p < 0.01.

To explore whether upregulation of *PDGFRA* in *IDH1*-mut AML may be linked to disrupted gene insulation due to CTCF binding site hypermethylation, we compared DNA methylation of *IDH1*-mut and *IDH1*-wt AML samples. Using the combined DNA methylation data from the SAL elderly AML (*n* = 79) and TCGA cohorts (*n* = 194), analyzed as described [5], we had previously identified elderly patients with *IDH1/2*-mut AML as distinct subgroup characterized by a global hypermethylation phenotype. Here, we further identified CpG hypermethylation within a CTCF-anchor region upstream of the *PDGFRA* gene locus in patients with *IDH1-*mut AML particularly (Fig. 2A, B). As an in vitro model for confirmation of gene expression data and analysis of DNA methylation and 3D DNA conformation, we generated a mutation knock-in of the *IDH1* p.R132H mutation in KG1a AML cells using CRISPR base editing. Successful heterozygous mutation knock-in was validated by sanger sequencing (Supplementary Fig. S3A) and 2-HG production of *IDH1*-mut clones was confirmed by 2-HG detection assay (Supplementary Fig. S3B). Using quantitative gene expression analysis (RT-qPCR), we confirmed significantly increased DNA methylation of the identified CTCF binding site in *IDH1*-mut cells compared to *IDH1*-wt cells (Fig. 2C). We further confirmed upregulated *PDGFRA* expression in *IDH1* p.R132H mutant cells (Fig. 2D). Both increased *PDGFRA* expression and *PDGFRA*-CTCF methylation was also observed in naïve KG1a cells treated with exogeneous 2-HG (Fig. 2C, D). We hypothesized that CpG hypermethylation may prevent CTCF binding and subsequently disrupt DNA loop formation and insulation of the *PDGFRA* gene, leading to *PDGFRA* upregulation. Therefore, we performed ChIP-qPCR of *IDH1*-wt and *IDH1*-mut KG1a cell clones after CTCF antibody pulldown. Quantification of CTCF-occupancy identified decreased CTCF binding by ~60% at the CTCF-anchor region upstream of the *PDGFRA* locus, suggesting altered DNA loop formation and subsequently disrupted insulation of the *PDGFRA* gene in *IDH1*-mut cells (Fig. 2E).

### Drug sensitivity and *PDGFRA* expression in treated *IDH1*-mut AML cells

**Fig. 3:**
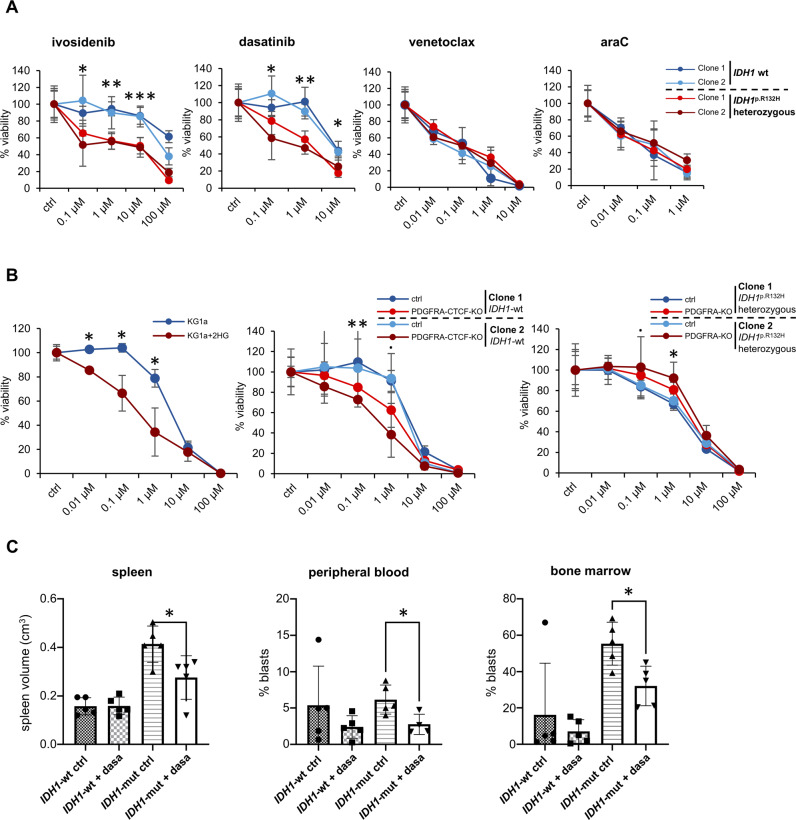
Drug sensitivity of *IDH1*-mut AML cells in vitro and in vivo. **A** Normalized cell viability (%) of *IDH1*-wt and *IDH1*-mut KG1a clones upon treatment with ivosidenib, dasatinib, venetoclax and araC (72 h) (2 clones, 3 replicates). Data represent mean ± SD. Student´s T-test, significance: *<0.05; **<0.01; ***<0.001. **B** Normalized cell viability (%) of 2-HG treated naïve KG1a cells, *IDH1*-mut/*PDGFRA*-KO cells and *IDH1*-wt/*PDGFRA*-CTCF-KO cells upon dasatinib treatment (72 h). Data represent mean ± SD. 2 clones, 3 replicates, Students T-test *p < 0.05; **p < 0.01. **C** Spleen volume, peripheral blood and bone marrow blast count (assessed by FACS for human CD45+ cells) in NSG mice injected with *IDH1*-wt versus *IDH1*-mut KG1a cells with daily dasatinib versus placebo treatment (mean ± SD). *n* = 5 per group, Mann–Whitney U test *p < 0.05; **p < 0.01.

To assess specific drug sensitivity of *IDH1*-mut AML cells in vitro, *IDH1*-mut and *IDH1*-wt KG1a cell clones were treated with the *IDH1*-mut specific inhibitor ivosidenib, the BCL2-inhibitor venetoclax, the standard chemotherapeutic agent cytarabine (araC) and the demethylating agent 5-azacytidine. Cell viability was assessed using WST assay. Mono-treatment with venetoclax and araC treatment reduced cell viability of both *IDH1*-mut and *IDH1*-wt cell clones (Fig. 3A). However, co-treatment with the ABCB1/MDR1/P-GP efflux pump inhibitor verapamil led to increased response of *IDH1*-mut cells to venetoclax compared to *IDH1*-wt cells (Supplementary Fig. S4), suggesting that blockade of the ABCB1/MDR1/P-GP efflux pump may be specifically relevant for the clinically benefit of Venetoclax treatment in *IDH1*-mut AML.

Confirming clinical data and the specificity of our cellular model, ivosidenib and 5-azacytidine treatment significantly reduced viability of *IDH1*-mut cells specifically and further resulted in decreased methylation of the *PDGFRA*-CTCF-anchor site as well as downregulation of *PDGFRA* gene expression (Fig. 3A, Supplementary Figs. S5, 6). This supports the functional mechanism of ivosidenib and 5-azacytidine in reducing 2-HG mediated DNA hypermethylation, resulting in restored DNA loop formation and subsequent gene expression. However, ivosidenib-mediated downregulation of *PDGFRA* gene expression was not durable and increased again after ivosidenib treatment pause in vitro (Supplementary Fig. S7).

### Increased sensitivity of *IDH1*-mut AML cells to tyrosine kinase inhibitor dasatinib

Ivosidenib resistance presents a major drawback and 5-azacytidine mediated DNA demethylation acts on a global and rather unspecific scale [27]. Therefore, we explored whether targeting *PDGFRA* with tyrosine kinase inhibitor (TKI) dasatinib may present an additional treatment option for *IDH1*-mut AML. *IDH1*-mut cell clones displayed increased sensitivity towards dasatinib compared to *IDH1*-wt clones, which was comparable to sensitivity towards *IDH1*-mut specific inhibitor ivosidenib (Fig. 3A). Cell viability data upon dasatinib and ivosidenib treatment were confirmed using FACS-based cell counting after drug treatment as an independent experimental approach (Supplementary Fig. S8). Confirming correlation of dasatinib sensitivity to upregulated *PDGFRA* expression, CRISPR knockout of *PDGFRA* reduced dasatinib sensitivity in *IDH1*-mut cell clones while CRISPR knockout of the *PDGFRA*-CTCF site and exogenous 2-HG treatment led to increased *PDGFRA* expression and dasatinib sensitivity in *IDH1*-wt cell clones and naïve KG1a cells (Fig. 3B, Supplementary Fig. S9A, B). As dasatinib also inhibits SRC kinases, we also assessed response to crenolanib as TKI without SRC inhibitory activity and observed a similarly increased response of *IDH1*-mut cell clones as to dasatinib in vitro (Supplementary Fig. S9C), supporting TKI treatment as potential novel treatment option.

### Additive effect of dasatinib in combination with ivosidenib

In vitro combination of ivosidenib and dasatinib resulted in an additive combination effect on *IDH1*-mut cells already at low ivosidenib concentrations, whereas in *IDH1*-wt cells combinations only appeared to be beneficial at the highest ivosidenib concentrations (Supplementary Fig. S10). To assess whether previous treatment with ivosidenib or 5-azacytidine may affect response to dasatinib in vitro, we treated *IDH1*-wt and *IDH1*-mut cell clones sequentially with ivosidenib and dasatinib or 5-azacytidine and dasatinib. Both pre-treatments did not reduce sensitivity of *IDH1*-mut cells to dasatinib (Supplementary Fig. S11), suggesting that the beneficial effect of dasatinib is not abrogated by previous ivosidenib or 5-azacytidine treatment. These data suggest dasatinib as potential candidate for specific treatment of patients with *IDH1*-mut AML.

### Increased dasatinib sensitivity in vivo and in a patient with *IDH1*-mut AML

To investigate dasatinib sensitivity in vivo, we generated an in vivo mouse model where *IDH1*-wt and *IDH1*-mut KG1a cells were injected into NSG mice that were subsequently treated with dasatinib or placebo. Dasatinib treatment of animals with *IDH1*-mut KG1a cells resulted in a significant decrease of spleen volume compared to placebo treatment (Fig. 3C). This treatment effect was not observed for animals with *IDH1*-wt KG1a cells. Additionally, leukemic blast count in peripheral blood and murine bone marrow were assessed by FACS analysis of human CD45-positive cells and revealed a significant decrease in leukemic burden in dasatinib-treated animals injected with *IDH1*-mut but not *IDH1*-wt KG1a cells (Fig. 3C).

**Fig. 4:**
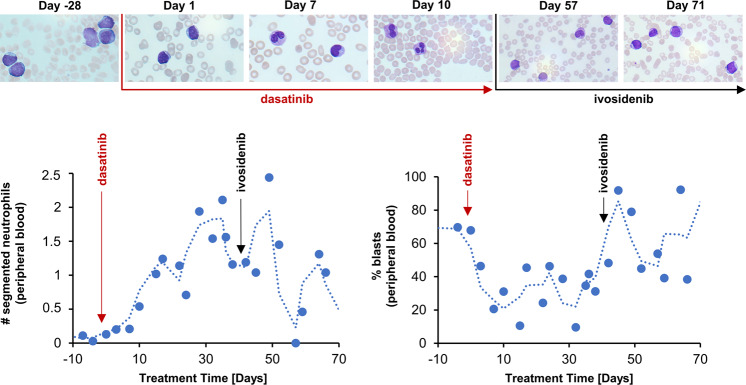
Segmented neutrophil and blast count in *IDH1*-mut AML patient after dasatinib treatment (case study). Top: Phase contrast images (40x) after Pappenheim staining, Bottom left: Segmented neutrophil count over time [days] under dasatinib (red arrow) and ivosidenib (black arrow) treatment, day 1 = first day of treatment with dasatinib as start of treatment cycle 4 after 3 previous treatment cycles, Bottom right: Blast count [%] over treatment time [days] under dasatinib and ivosidenib treatment.

In addition, in a single-case study of a patient with refractory AML with *IDH1* mutation, dasatinib treatment increased the number of segmented neutrophils while the number of leukemic blasts decreased (Fig. 4). Performance status of the patient improved dramatically and treatment was administered in an out-patient setting. However, response to dasatinib was not durable and refractory relapse was noted after 2 months. Subsequent treatment with Ivosidenib failed to induce meaningful disease control (Fig. 4).

## Discussion

In this study, we identified receptor tyrosine kinase *PDGFRA* as upregulated oncogene in AML with *IDH1* p.R132H mutation. Furthermore, we provide first evidence that *PDGFRA* upregulation occurs as a result of disrupted DNA loop formation and subsequent loss of gene insulation due to CpG hypermethylation within a CTCF-anchor region upstream of the *PDGFRA* gene locus in AML. *IDH* mutations have previously been associated with global DNA hypermethylation in AML as a result of the generation of the oncometabolite 2-HG, which inhibits TET-mediated DNA demethylation [5, 9]. When located within promoter regions of genes involved in cell differentiation, CpG hypermethylation has been shown to lead to a block of myeloid differentiation [7]. However, a role of 2-HG mediated hypermethylation in oncogene activation due to disrupted DNA architecture has not been shown in the context of *IDH1*-mut AML so far.

Aberrant 3D DNA architecture has generally been reported for different malignancies when compared to healthy controls [13, 28–33]. In the context of developmental disorders, Melo et al. identified complex genomic rearrangements and TAD-shuffling, which was associated with phenotypic changes. In leukemia, Hyle et al. showed that CTCF depleting results in loss of *MYC* expression by disrupted *MYC* promotor-enhancer interaction in B-cell precursor acute lymphoblastic leukemia (BCP-ALL) [33]. With regard to epigenetic mutations, changes in 3D architecture and subsequently altered gene expression has been reported as well [31, 34, 35]. In AML, disrupted CTCF binding at the CTCF binding site between the *HOXA7* and *HOXA9* genes was reported to prevent DNA looping and alter expression of posterior *HOXA* genes [14]. In *IDH1*-mutant AML, Wilson et al. recently investigated differentially methylated regions (DMR) with regard to enhancer association and identified high enrichment for enhancers in hypermethylated *IDH1*-mut specific DMRs [36]. However, an association to differential gene expression of the correlating genes could not be identified. Our data link disrupted 3D DNA architecture in *IDH1*-mut AML to upregulated oncogene expression, providing novel insights into 3D DNA alterations as additional leukemogenesis-driving mechanism in AML.

Here we show that upregulation of tyrosine kinase *PDGFRA* is associated with altered 3D DNA conformation in *IDH1*-mut AML and therefore may represent a novel therapeutic target in *IDH1*-mut AML. *PDGFRA* is involved in cell proliferation, migration and invasion, cell survival and chemotaxis [37]. In the Network of Cancer Genes (NGC) project it has also been classified as driver gene with oncogenic potential and its overexpression has been reported in breast cancer, ovarian cancer and thyroid cancer [38–40]. Yet, activation of *PDGFRA* expression in connection with altered DNA conformation as potential leukemogenic mechanism in *IDH1*-mut AML has not been suggested. We provide evidence for decreased CTCF binding at the CTCF-anchor region upstream of *PDGFRA* due to increased DNA methylation, suggesting disrupted DNA loop formation and insulation and subsequent upregulation of *PDGFRA* expression. Upregulated *PDGFRA*-CTCF methylation and *PDGFRA* expression was also confirmed upon exogenous treatment with 2-HG, confirming 2-HG mediated CTCF hypermethylation as mechanism of action upregulating *PDGFRA*. This finding is supported by the work from the B. Bernstein research group showing disrupted DNA looping and subsequent activation of *PDGFRA* expression in *IDH1*-mut glioma [12]. Our finding of upregulated *PDGFRA* expression in connection with altered DNA looping in *IDH1*-mut AML now extends this mechanism of action beyond the context of glioma. As observed in clinical data of *IDH1*-mut AML patients, *IDH1*-mut glioma patients with high *PDGFRA* expression also showed significantly worse survival rates, supporting a strong clinical relevance of this mechanism. These data strengthen the hypothesis that the correlation between *IDH1* mutation-dependent DNA conformational changes and upregulated *PDGFRA* expression may be a more global mechanism across different cancer types.

Concerning treatment options for *IDH1*-mut AML, the small-molecule inhibitor ivosidenib presents an effective therapeutic agent, leading to remissions in *IDH1*-mut AML patients [41]. However, responses are often not durable and relapse and drug resistance present a major problem associated with ivosidenib monotherapy, pointing out the importance of identifying novel treatment strategies [11]. For this purpose, the identification of tyrosine kinase *PDGFRA* as novel target may not only be of biological or prognostic [42] but also of therapeutic relevance.

Here, we provide evidence that treatment with tyrosine kinase inhibitor dasatinib may present a novel treatment option for AML patients with *IDH1* p.R132H mutation. While *PDGFRA* knockout reduced dasatinib sensitivity in *IDH1*-mut clones, knockout of the *PDGFRA*-CTCF site and exogenous 2-HG treatment increased dasatinib sensitivity in *IDH1*-wt clones and naïve KG1a cells, confirming the specific correlation of dasatinib response to *PDGFRA* expression. The antileukemic effect of dasatinib was confirmed in an in vivo mouse model of *IDH1*-wt/-mut KG1a and also observed in a single patient with *IDH1*-mut AML, supporting dasatinib as potential treatment option for *IDH1*-mut AML. Additionally, our in vitro data also provide a base for the combination of dasatinib and ivosidenib as potential treatment approach.

With regard to combinational therapies, clinical trials have shown promising effects of combination of the demethylating agent 5-azacytidine or BCL2-inhibitor venetoclax with ivosidenib [43, 44]. In our cellular model, the addition of ABCB1/MDR1/P-GP efflux pump inhibitor verapamil led to increased venetoclax response of *IDH1*-mut cells while venetoclax monotherapy resulted in a similar response of *IDH1*-mut and *IDH1*-wt KG1a cells. This suggests that inhibition of the ABCB1/MDR1/P-GP efflux pump may be specifically relevant in this *IDH1*-mut KG1a model for the observation of mutation-specific sensitivity differences as reported previously [44].

In addition, increased sensitivity of *IDH1*-mut versus *IDH1*-wt cells to 5-azacytidine could be confirmed, which supports data from recent clinical trials suggesting 5-azacytidine as combinational partner for ivosidenib treatment of patients with *IDH1*-mut AML [43]. However, as 5-azacytidine treatment leads to DNA demethylation on a global scale rather than specific demethylation of the *PDGFRA*-CTCF site [27], Treatment with dasatinib as TKI may add a more targeted treatment option for *IDH1*-mut AML specifically.

The suggestion of dasatinib as *IDH1* mutant-specific treatment is supported by data from Tavor et al. [45] revealing a significant enrichment of samples with *IDH1* mutations (33.3%) versus *IDH1*-wt AML samples (14.3%) among those responding to dasatinib ex vivo (hypergeometric test, *p* = 0.022). Tavor and colleagues also reported an enrichment of *FLT3-ITD* and *IDH2*-mut samples among dasatinib responders in this study. However, our analysis of the underlying RNA-Seq data (BeatAML) [15] revealed no significant difference in expression of *PDGFRA* in *IDH2*-mut versus *IDH1*-wt AML samples (Supplementary Fig. S1A). This suggests that the underlying mechanism of *IDH2* mutant-specific sensitivity to dasatinib may differ from our observed *IDH1* mutant-specific dasatinib sensitivity and may be related to other dasatinib targets than *PDGFRA*. This would be compatible with the data from Wilson et al. reporting differences in global hypermethylation patterns of *IDH1*-mut and *IDH2*-mut AML [36]. Specific promoter methylation differences may then translate into different downstream gene expression and pathway activation and may therefore provide a possible explanation for a different underlying mechanism of dasatinib response.

*IDH1*-mut specificity of dasatinib is further supported by the reduced leukemic blast count that was observed in a patient with refractory *IDH1*-mut AML after dasatinib treatment. As ivosidenib or 5-azacytidine pre-treatment did not abrogate the beneficial effect of dasatinib in *IDH1*-mut AML cells and as the combination of dasatinib and ivosidenib appeared to be beneficial, our data suggest dasatinib as novel treatment option, also in combinations, for patients with *IDH1* p.R132H mutant AML.

In conclusion, our data suggest that *IDH1* p.R132H mutation induces changes in the 3D DNA architecture leading to alteration of long-range gene interactions in AML. We show that methylation-dependent disruption of CTCF binding at the CTCF-anchor upstream of *PDGFRA* leads to upregulated *PDGFRA* expression. As *PDGFRA* overexpression correlated with decreased survival of patients with *IDH1*-mutant AML, we reason that treatment with TKI dasatinib may present a novel therapeutic option for improved treatment for patients with *IDH1* p.R132H mutant AML. This may be of particular interest for elderly patients with mutant *IDH1*, which present a large proportion of patients and show a particularly high *IDH* mutation frequency and, so far, poor outcome with high resistance and relapse rates.

## Supplementary information

Supplementary Material

Supplementary Table 4

## Data Availability

The datasets analyzed during the current study are available at the dbGaP repository with the study ID is 30641 and accession ID is phs001657 or at the NCBI Gene Expression Omnibus repository (GSE146173, GSE6891, GSE86409). All further data generated during this study are included in this published article and its Supplementary Information files.

## References

[1] Montalban-Bravo G, DiNardo CD (2018). The role of IDH mutations in acute myeloid leukemia. Future Oncol.

[2] Shallis RM, Wang R, Davidoff A, Ma X, Zeidan AM (2019). Epidemiology of acute myeloid leukemia: Recent progress and enduring challenges. Blood Rev.

[3] Bullinger L, Döhner K, Döhner H (2017). Genomics of Acute Myeloid Leukemia Diagnosis and Pathways. J Clin Oncol.

[4] Döhner H, Estey E, Grimwade D, Amadori S, Appelbaum FR, Büchner T (2017). Diagnosis and management of AML in adults: 2017 ELN recommendations from an international expert panel. Blood..

[5] Silva P, Neumann M, Schroeder MP, Vosberg S, Schlee C, Isaakidis K (2017). Acute myeloid leukemia in the elderly is characterized by a distinct genetic and epigenetic landscape. Leukemia..

[6] Medeiros BC, Fathi AT, DiNardo CD, Pollyea DA, Chan SM, Swords R (2017). Isocitrate dehydrogenase mutations in myeloid malignancies. Leukemia..

[7] Al-Khallaf H (2017). Isocitrate dehydrogenases in physiology and cancer: biochemical and molecular insight. Cell Biosci.

[8] Molenaar RJ, Maciejewski JP, Wilmink JW, van Noorden CJF (2018). Wild-type and mutated IDH1/2 enzymes and therapy responses. Oncogene..

[9] Figueroa ME, Abdel-Wahab O, Lu C, Ward PS, Patel J, Shih A (2010). Leukemic IDH1 and IDH2 mutations result in a hypermethylation phenotype, disrupt TET2 function, and impair hematopoietic differentiation. Cancer Cell.

[10] Golub D, Iyengar N, Dogra S, Wong T, Bready D, Tang K (2019). Mutant Isocitrate Dehydrogenase Inhibitors as Targeted Cancer Therapeutics. Front Oncol.

[11] Donker ML, Ossenkoppele GJ (2020). Evaluating ivosidenib for the treatment of acute myeloid leukemia. Expert Opin Pharmacother.

[12] Flavahan WA, Drier Y, Liau BB, Gillespie SM, Venteicher AS, Stemmer-Rachamimov AO (2015). Insulator dysfunction and oncogene activation in IDH mutant gliomas. Nature..

[13] Yang L, Chen F, Zhu H, Chen Y, Dong B, Shi M (2021). 3D genome alterations associated with dysregulated HOXA13 expression in high-risk T-lineage acute lymphoblastic leukemia. Nat Commun.

[14] Luo H, Wang F, Zha J, Li H, Yan B, Du Q (2018). CTCF boundary remodels chromatin domain and drives aberrant HOX gene transcription in acute myeloid leukemia. Blood..

[15] Tyner JW, Tognon CE, Bottomly D, Wilmot B, Kurtz SE, Savage SL (2018). Functional genomic landscape of acute myeloid leukaemia. Nature..

[16] Bamopoulos SA, Batcha AMN, Jurinovic V, Rothenberg-Thurley M, Janke H, Ksienzyk B (2020). Clinical presentation and differential splicing of SRSF2, U2AF1 and SF3B1 mutations in patients with acute myeloid leukemia. Leukemia..

[17] Verhaak RG, Wouters BJ, Erpelinck CA, Abbas S, Beverloo HB, Lugthart S (2009). Prediction of molecular subtypes in acute myeloid leukemia based on gene expression profiling. Haematologica..

[18] Komor AC, Kim YB, Packer MS, Zuris JA, Liu DR (2016). Programmable editing of a target base in genomic DNA without double-stranded DNA cleavage. Nature..

[19] Bustin SA, Benes V, Garson JA, Hellemans J, Huggett J, Kubista M (2009). The MIQE guidelines: minimum information for publication of quantitative real-time PCR experiments. Clin Chem.

[20] Love MI, Huber W, Anders S (2014). Moderated estimation of fold change and dispersion for RNA-seq data with DESeq2. Genome Biol.

[21] Peake K, Manning J, Lewis CA, Barr C, Rossi F, Krieger C. Busulfan as a myelosuppressive agent for generating stable high-level bone marrow chimerism in mice. J Vis Exp. 2015;e52553. 10.3791/52553 PMC4401399 25867947

[22] Saland E, Boutzen H, Castellano R, Pouyet L, Griessinger E, Larrue C (2015). A robust and rapid xenograft model to assess efficacy of chemotherapeutic agents for human acute myeloid leukemia. Blood Cancer J.

[23] Wilkinson FL, Sergijenko A, Langford-Smith KJ, Malinowska M, Wynn RF, Bigger BW (2013). Busulfan conditioning enhances engraftment of hematopoietic donor-derived cells in the brain compared with irradiation. Mol Ther.

[24] Schewe DM, Lenk L, Vogiatzi F, Winterberg D, Rademacher AV, Buchmann S (2019). Larotrectinib in TRK fusion-positive pediatric B-cell acute lymphoblastic leukemia. Blood Adv.

[25] Schewe DM, Alsadeq A, Sattler C, Lenk L, Vogiatzi F, Cario G (2017). An Fc-engineered CD19 antibody eradicates MRD in patient-derived MLL-rearranged acute lymphoblastic leukemia xenografts. Blood..

[26] Lenk L, Winterberg D, Vogiatzi F, Laqua A, Spory L, Mayar A (2022). Preclinical Evidence for the Efficacy of CD79b Immunotherapy in B-cell Precursor Acute Lymphoblastic. Leuk Hemasphere.

[27] Grövdal M, Karimi M, Tobiasson M, Reinius L, Jansson M, Ekwall K (2014). Azacitidine induces profound genome-wide hypomethylation in primary myelodysplastic bone marrow cultures but may also reduce histone acetylation. Leukemia..

[28] Melo US, Schöpflin R, Acuna-Hidalgo R, Mensah MA, Fischer-Zirnsak B, Holtgrewe M (2020). Hi-C Identifies Complex Genomic Rearrangements and TAD-Shuffling in Developmental Diseases. Am J Hum Genet.

[29] Hnisz D, Weintraub AS, Day DS, Valton AL, Bak RO, Li CH (2016). Activation of proto-oncogenes by disruption of chromosome neighborhoods. Science..

[30] Grimmer MR, Costello JF (2016). Cancer: Oncogene brought into the loop. Nature.

[31] Zhang X, Jeong M, Huang X, Wang XQ, Wang X, Zhou W (2020). Large DNA Methylation Nadirs Anchor Chromatin Loops Maintaining Hematopoietic Stem Cell Identity. Mol Cell.

[32] Flavahan WA, Drier Y, Johnstone SE, Hemming ML, Tarjan DR, Hegazi E (2019). Altered chromosomal topology drives oncogenic programs in SDH-deficient GISTs. Nature..

[33] Hyle J, Zhang Y, Wright S, Xu B, Shao Y, Easton J (2019). Acute depletion of CTCF directly affects MYC regulation through loss of enhancer–promoter looping. Nucleic Acids Res.

[34] Ghasemi R, Struthers H, Wilson ER, Spencer DH (2021). Contribution of CTCF binding to transcriptional activity at the HOXA locus in NPM1-mutant AML cells. Leukemia..

[35] Donaldson-Collier MC, Sungalee S, Zufferey M, Tavernari D, Katanayeva N, Battistello E (2019). EZH2 oncogenic mutations drive epigenetic, transcriptional, and structural changes within chromatin domains. Nat Genet.

[36] Wilson ER, Helton NM, Heath SE, Fulton RS, Payton JE, Welch JS, et al. Focal disruption of DNA methylation dynamics at enhancers in IDH-mutant AML cells. Leukemia. 2021;36:935–45. 10.1038/s41375-021-01476-y PMC8979817 34873300

[37] Ding H, Wu X, Boström H, Kim I, Wong N, Tsoi B (2004). A specific requirement for PDGF-C in palate formation and PDGFR-alpha signaling. Nat Genet.

[38] Li Q, Li M, Zheng K, Tang S, Ma S (2021). Expression pattern analysis and drug differential sensitivity of cancer-associated fibroblasts in triple-negative breast cancer. Transl Oncol.

[39] Lopez-Campistrous A, Adewuyi EE, Williams DC, McMullen TPW. Gene expression profile of epithelial-mesenchymal transition mediators in papillary thyroid cancer. Endocrine. 2020;72:452-61. 10.1007/s12020-020-02466-3 32914379

[40] Zhang T, Zhang L, Li F Integrative network analysis identifies potential targets and drugs for ovarian cancer. BMC Med Genomics. 2020;13:132. 10.1186/s12920-020-00773-2 PMC7504661 32958005

[41] Popovici-Muller J, Lemieux RM, Artin E, Saunders JO, Salituro FG, Travins J (2018). Discovery of AG-120 (Ivosidenib): A First-in-Class Mutant IDH1 Inhibitor for the Treatment of IDH1 Mutant Cancers. ACS Med Chem Lett.

[42] Wang F, Morita K, DiNardo CD, Furudate K, Tanaka T, Yan Y (2021). Leukemia stemness and co-occurring mutations drive resistance to IDH inhibitors in acute myeloid leukemia. Nat Commun.

[43] DiNardo CD, Stein AS, Stein EM, Fathi AT, Frankfurt O, Schuh AC (2021). Mutant Isocitrate Dehydrogenase 1 Inhibitor Ivosidenib in Combination With Azacitidine for Newly Diagnosed Acute Myeloid Leukemia. J Clin Oncol.

[44] DiNardo CD, Tiong IS, Quaglieri A, MacRaild S, Loghavi S, Brown FC (2020). Molecular patterns of response and treatment failure after frontline venetoclax combinations in older patients with AML. Blood.

[45] Tavor S, Shalit T, Ilani NC, Moskovitz Y, Livnat N, Groner Y (2020). Dasatinib response in acute myeloid leukemia is correlated with FLT3/ITD, PTPN11 mutations and a unique gene expression signature. Haematologica..

[46] Rao SS, Huntley MH, Durand NC, Stamenova EK, Bochkov ID, Robinson JT (2014). A 3D map of the human genome at kilobase resolution reveals principles of chromatin looping. Cell.

