# Supplementary material for: Isocitrate dehydrogenase 1 mutation drives leukemogenesis by PDGFRA activation due to insulator disruption in acute myeloid leukemia (AML)
Source: Leukemia. 2022 Nov 21;37(1):134–42. doi: 10.1038/s41375-022-01751-6 (PMC9883162; doi:10.1038/s41375-022-01751-6)
Supplement: Supplementary file 1 — Supplementary Material [file 41375_2022_1751_MOESM1_ESM.pdf]

Supplementary Material

Isocitrate dehydrogenase 1 mutation drives leukemogenesis by PDGFRA activation due to insulator disruption in acute myeloid leukemia (AML)

Supplementary Methods

CRISPR base editing

Here, a fusion enzyme of catalytically inactive Cas9 (dCas9) and cytidine deaminase introduces a C-to-T point mutation within the activity window of cytidine deaminase (positions 4 to 8, counting from the end distal to the PAM). As result, a substitution from G-to-A is introduced in the complementary strand, transforming codon 132 in the *IDH1* gene from arginine to histidine. Cells were co-transfected with one plasmid encoding the dCas9/base-editor and GFP (#112099, Addgene, Watertown, MT, United States) and another plasmid containing the *IDH1* p.R132H sgRNA and a neomycin resistance gene (#41824, Addgene). 24 h prior transfection, cells were cultured in antibiotic-free RPMI + 20% FBS. Transfection was performed by electroporation using the NEON electroporation system (settings: 1600 V / 10 ms / 3 pulses). After 24 h, GFP+ cells were sorted into a bulk and selected using Geneticin/G418 (Thermo Fisher Scientific, Waltham, MA, USA) for 5 days, followed by single cell cloning. After 5 weeks, Terra PCR (TakarBio, Goteborg, Sweden) was performed to screen for *IDH1*-wt or *IDH1*-mut clones.

CRISPR knockout

24 h prior transfection, cells were cultured in antibiotic-free RPMI + 20% FBS. Transfection was performed by electroporation using the NEON electroporation system (settings: 1600 V / 10 ms / 3 pulses). 48 h after electroporation of synthetic sgRNAs and Cas9 RNP,DNA and RNA of the generated CRISPR bulk cells was extracted for assessment of *PDGFRA* expression and *PDGFRA*-CTCF methylation as well as dasatinib sensitivity. Sanger sequencing primers and sgRNAs for *PDGFRA* knockout and *PDGFRA*-CTCF knockout (Flavahan et al. 2015) are shown in suppl. Table 1. *PDGFRA*-CTCF knockout primers are equivalent to RT-qPCR primers for assessment of methylation (suppl. Table 1).

Differential gene expression and survival analysis

To identify upregulated oncogenes in *IDH1*-mut AML, differential gene expression was analyzed in two independent datasets, the BeatAML RNA-Seq dataset with n = 562 patients in total and n = 264 patients with annotated *IDH1* status and a microarray dataset by Verhaak et al. 2009 with n = 247 patients. Analysis was performed in R v4.0.3 with VST values generated from RNA-Seq raw counts using DESeq2. Gene Ontology enrichment analysis was performed with ShinyGO software v0.75 using GO Molecular Function.

To investigate the clinical relevance of identified oncogenes, overall survival of patients with high/low oncogene expression with/without *IDH1* mutation was analyzed in two independent clinical datasets (BeatAML and Bamopoulos et al. 2020, n = 246). Kaplan-Meier survival analysis was performed in R v4.0.3 using the R package “survival” and “survfit” functions, displaying the log-rank test p-value. Both DESeq2 and survfit account for data variance and do not require normal distribution. An overview of all datasets analyzed is provided in suppl. Table 3.

DNA Methylation and 3D DNA conformation data

CpG methylation within the CTCF anchor upstream of *PDGFRA* was compared between *IDH1*-wt and *IDH1*-mut AML patients using Wilcoxon test with Benjamini Hochberg FDR correction on β-logit2 transformed M-values. Human CTCF ChIP-Seq data and CHIA-PET data for long-range chromatin interactions for the myeloid leukemia cell line K562 were retrieved from the ENCODE data repository site (http://genome.ucsc.edu/ENCODE/) and visualized using IGV Genome Browser on hg38 (https://igv.org/app/).

RNA, cDNA, RT-qPCR

RNA was extracted using the RNEasy Plus Mini extraction kit (Qiagen, Venlo, Netherlands) and reverse transcribed in cDNA using the iScript cDNA synthesis kit (Bio Rad, Hercules, CA, USA). Quantitative Real-Time PCR (RT-qPCR) was performed with 10 ng cDNA per reaction using Fast Start Essential DNA Green Master Mix (Roche, Basel, Switzerland) on a Roche LightCycler LC96 system. All RT-qPCR primers were designed using NCBI Primer3 software and are listed in suppl. Table 1.

Methylation-sensitive restriction digest

To assess CTCF methylation, genomic DNA from *IDH1*-wt and -mut cells was extracted using All Prep DNA/RNA Mini kit (Qiagen) and 1000 ng DNA was digested with 10 units of methylation-sensitive HinP1I restriction enzyme (New England Biolabs (NEB), Ipswich, MA, USA) for 15 minutes at 37°C followed by heat inactivation at 65°C. HinP1I recognizes the restriction site GCGC, which is present within the CTCF binding site upstream of the *PDGFRA* gene locus. If genomic DNA is methylated, cleavage of HinP1I is blocked and the *PDGFRA*-CTCF site can be amplified and quantified using RT-qPCR. RT-qPCR primers for assessment of methylation (Flavahan et al. 2015) are listed in suppl. Table 1.

Assessment of CTCF binding using ChIP-qPCR

For assessment of CTCF binding in *IDH1*-wt and *IDH1*-mut KG1a cell clones using ChIP-qPCR, nuclei of 10 million formaldehyde-crosslinked cells were lysed and DNA was sonicated on a Covaris (S-Series 220). After sonication, DNA was immunoprecipitated overnight at 4°C against a CTCF antibody (Millipore, Burlington, MA, USA), reverse crosslinking with proteinase K (NEB) overnight at 65˚C and purified using the Zymo Research ChIP DNA Clean and Concentrator Kit (BIOSITE-D5205). ChIP-qPCR was performed and analyzed as described in Flavahan et al. 2015 with PSMB1 as reference gene unaffected by DNA loop formation. RT-qPCR primers for assessment of CTCF binding are listed in suppl. Table 1.

In vitro drug sensitivity screenings, 2-HG detection and treatment

To assess drug sensitivity, 30,000 cells were treated with ivosidenib (ivo), dasatinib (dasa), venetoclax (vnx), cytarabine (araC), 5-azacytidine (aza) or crenolanib (cre) in a 96 well plate for 72 h. Concentrations ranges of 0.1-100 µM (ivo, cre), 0.1-10 µM (dasa, vnx) and 0.01-1 µM (araC) were chosen with 1:10 dilution steps or 0.08-50 µM (aza) in 1:5 dilution steps. Therapeutic agents were purchased from Selleckchem (Houston, TX, USA). After 72 h treatment, viability was assessed as absorbance at 450 nm using WST assay on a Synergy HTX multi-mode reader system (BioTek, Winooski, VT, USA). Percent viability was calculated relative to untreated cells. For combinational treatments, synergistic effects were assessed using Combenefit analysis software and the Loewe additivity model. For sequential treatments, cells were either treated for 72 h with 5-azacytidine, ivosidenib or dasatinib alone or for 36 h with 5-azacytidine or ivosidenib followed by 36 h of dasatinib. Cell viability after treatment was assessed as stated above. 2-HG production was measured in conditioned media from *IDH1*-wt and *IDH1*-mut cell clones were cultured for 48 h using the D-2-Hydroxyglutarate (D2HG) Assay Kit (Sigma-Aldrich, MO, USA), according to the manufacture's protocol. To assess the effect of exogenous 2-HG on *PDGFRA* expression, *PDGFRA*-CTCF methylation and dasatinib response, KG1a cells were treated with 20 mM 2-HG (Sigma-Aldrich, MO, USA) for 24 h followed by RNA/DNA extraction or dasatinib sensitivity screening by WST assay.

In vivo assessment of dasatinib sensitivity

For this study, a total of n = 20 8-weeks old female SCID/NOD/IL2R$_\gamma$null (NSG) mice (Jackson Laboratories, Maine, USA) have been used with the group size of n = 5. Sample size was assessed by G*Power using Fisher exact test based on observed *in vitro* effect

size and approved by the Ministerium für Energiewende, Landwirtschaft, Umwelt, Natur und Digitalisierung (MELUND), Schleswig-Holstein, Germany. Two million *IDH1*-wt or *IDH1*-mut KG1a cells were injected intravenously into busulfan-pretreated NSG-mice (Peake et al. 2015, Saland et al. 2015, Wilkinson et al. 2013, Schewe et al. 2017, Lenk et al. 2022) and animals were treated daily with 50 mg/kg dasatinib or vector control (n = 5 each) by oral gavage (Tavor et al. 2020, Schewe et al. 2019) in a non-randomized manner. The therapy was initiated on day 8 (after leukemic engraftment) (Schewe et al. 2019). All animals were sacrificed after 56 days when the first control group animals of both KG1a groups had shown clinical signs of leukemia such as ataxia and weight loss (Schewe et al. 2019). Leukemic engraftment quantified by organ-specific leukemic blast counts was assessed by FACS analysis of human CD45-positive cells in peripheral blood and bone marrow. Statistically significant differences in blast counts and spleen volume were assessed in R using Mann-Whitney U test.

Supplementary Table 1: Sequences of CRISPR sgRNA for *IDH1* p.R132H mutation knock-in, **Sanger sequencing primers and** RT-qPCR primers.

Supplementary Table 1*: Summary of all primer sequences (5´ to 3´) used for analysis of PDGFRA expression, PDGFRA-CTCF methylation and PDGFRA-CTCF occupancy as well as CRISPR sgRNA sequence and Sanger sequencing primers.*

**Method**	**Primer**	**Sequence 5´-> 3´**
**CRISPR**		
IDH1 base editing	IDH1-R132H sgRNA	GCATGACGACCTATGATGAT
PDGFRA knockout	PDGFRA sgRNA 1	GGATAGAGGGTAATGAAAGC
	PDGFRA sgRNA 2	TGAAGAAGAGAGCTCCGATG
PDGFRA-CTCF knockout	PDGFRA-CTCF sgRNA	CCACAGATAATGCAGCTAGA
**Sanger Sequencing IDH1**		
IDH1 base editing	IDH1_seq_F	AGCTCTATATGCCATCACTGC
	IDH1_seq_R	GCATTTCTCAATTTCATACCTTGCT
PDGFRA knockout	PDGFRA_F	TGTCGGGATGAGACTGTCCT
	PDGFRA_R	AAGAAGCTTGGTCCTGGAGAC
**RT-qPCR**		
Gene expression quantification	Ref_HPRT1_qF	AGGATTTGGAAAGGGTGTTTATTC
	Ref_HPRT1_qR	CAGAGGGCTACAATGTGATGG
	PDGFRA_qF	TTGAAGGCAGGCACATTTACA
	PDGFRA_qR	GCGACAAGGTATAATGGCAGAAT
PDGFRA-CTCF occupancy	Ref_PSMB1-ctcf_qF	CCTTCCTAGTCACTCAGTAA
	Ref_PSMB1-ctcf_qR	CAGTGTTGACTCATCCAG
	PDGFRA-ctcf_qF	GTCACAGTAGAACCACAGAT
	PDGFRA-ctcf_qR	TAAGTATACTGGTCCTCCTC
PDGFRA-CTCF methylation	PDGFRA-HinP1I-qF	CACGTGAGCTGAATTGTGCC
	PDGFRA-HinP1I-qR	GGTCCTCCTCTCCCAAGACT

Supplementary Table 2: Executive summary of single case patient with refractory AML with *IDH1* mutation.

Supplementary Table 2*: Executive summary of single case patient with refractory AML with IDH1 mutation.*

Relapsed Acute Myeloblastic Leukemia

- Hematology at initial diagnosis (peripheral blood): WBC 104/nl, Blast 60%, Hb 9,5 g/dl, Plt 9/nl, LDH 1288 U/l.
- Cytology (Bone marrow): AML FAB M2, Infiltration 80%
- Cytogenetics: 46, XY, der(9)t(8;9)(q23;q34); partial Trisomy 8q
- Molecular Genetics at initial diagnosis: CEPBA (TAD 1 bp Ins, bZIP 46 bp Ins) biallelic mutated
- Molecular Genetics (Month 22): IDH1 pos, CEBPA pos., DNMT3A pos., JAK2 pos.
- WHO classification: AML with biallelic CEBPA mutation
- ELN classification: favourable
- Family history: none
- Donor search: MUD available

Patient trajectory

**M0**	**Initial diagnosis**
	Induction I+II - *Daunorubicin/Cytarabine* (3+7)
M2-M4	Consolidation I-III (*Cytarabine* 18 g/m²)
M4	Remission assessment: complete remission (CR-1)
**M9**	**Relapse #1**
	Salvage chemotherapy (FLAG-IDA)
	Remission assessment: blast persistence
M10	*Azacytidine (Vidaza)/Venetoclax* (V^2)
M12	Remission assessment: complete remission (CR-2)
M13	Allogeneic hematopoietic stem cell transplantation from matched unrelated donor
	Conditioning: *Fludarabine, Thiotepa, Melphalan* (FTM) und ATG
M15	Remission assessment: complete remission
**M19**	**Relapse #2**
	Cytology (Bone marrow): blast infiltration 60%
	Molecular genetics: IDH1 mutated (non-Hotspot, c1180A>G),
M20	2 cycles *Vidaza/Venetoclax*
M22	3 x donor lymphocyte infusions
M24	Remission assessment: refractory disease (blast infiltration 42%)
	Inclusion to ARMADA study (HAM +/- CPI-613)
	Treatment according to control arm (HAM)
**M25**	**Remission assessment: refractory disease**
M26	Application for compassionate use Ivosidenib
	Off-Label treatment with ***Dasatinib*** 140 mg/d
	**Remission assessment: stable disease**
	Complication: Erythema exsudativum multiforme CTCAE III
	Dasatinib withdrawn
M27	Approval compassionate use ***Ivosidenib***
	Treatment initiation Ivosidenib
**M29**	**Remission assessment: refractory disease**
	Exitus letalis (Cause of death: intracranial hemorrhage)

Supplementary Table 3: Summary of datasets analyzed in this study.

Supplementary Table 3*: Summary of datasets analyzed in this study. Overview of all datasets analyzed with regard to gene expression, survival and methylation.*

Dataset	# patients	Gene expression		Survival	Methylation
		RNA-Seq	Affymetrix Microarray		Infinity hm 450k
Tyner et al., 2018 (BeatAML)	n = 562	**x**		**x**	
Bamopoulos et al., 2020	n = 246			**x**	
Verhaak et al., 2009	n = 247		**x**		
Silva et al., 2017 + TCGA (LAML)	n = 273				**x**

Supplementary Table 4: Upregulated genes in *IDH1*-mut AML (BeatAML dataset).

Supplementary Table 4*: Upregulated genes in IDH1-mut AML (BeatAML dataset). Table displays baseMean, log2FoldChange, lfcSE, stat, pvalue, padj and gene name of 132 upregulated genes in IDH1-mut AML with log2FoldChange cut-off = 1 and padj cut-off < 0.05. Differential genes expression was analyzed using DESeq2.*

Table is provided as separate .xlsx file

Supplementary Figure S1: *PDGFRA* expression in patients with *IDH1*-wt/mut versus *IDH2*-wt/mut AML (BeatAML data) and *IDH1*-wt/mut AML RNA microarray data (Verhaak 2008)

A ***PDGFRA*** **expression in** ***IDH1*****-wt/mut versus** ***IDH2*****-wt/mut AML (BeatAML)**

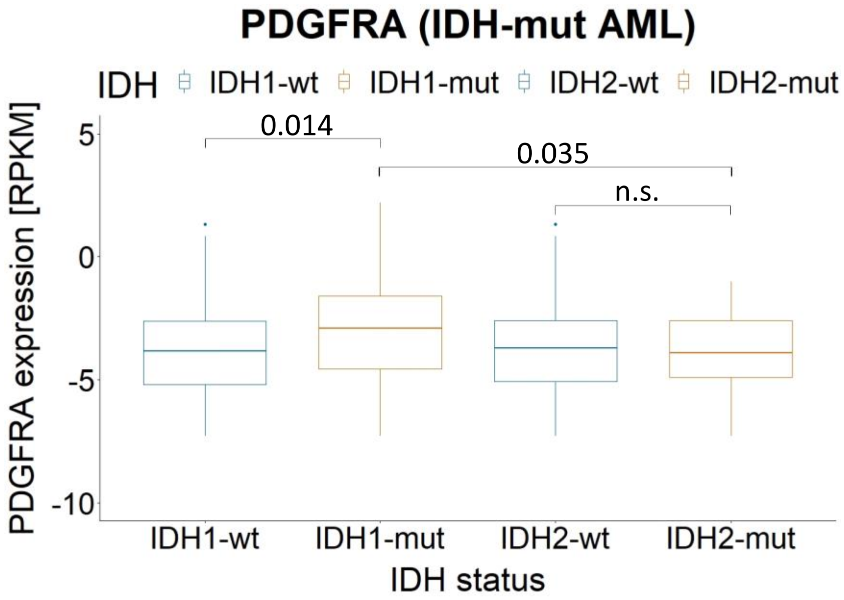

B ***PDGFRA*** **expression in RNA microarray data (Verhaak 2008) from patients with** ***IDH1*****-mut versus** ***IDH1*****-wt AML**

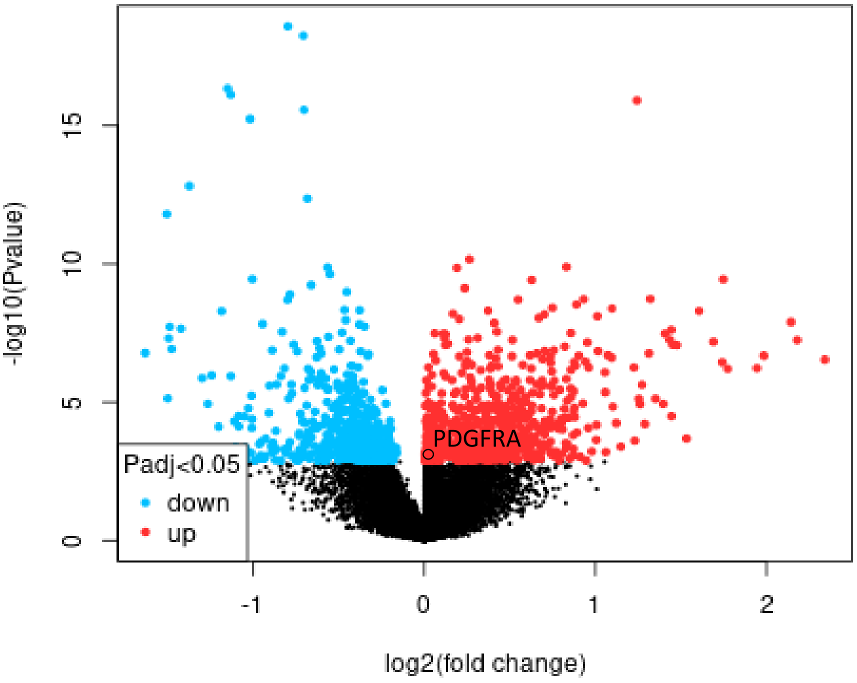

Supplementary Figure S1*: A) Higher expression of PDGFRA (RPKM) in IDH1-mut vs. IDH2-mut AML (data: BeatAML. IDH1-mut: n = 28, IDH1-wt: n = 153, IDH2-mut: n = 33, IDH2-wt: n = 143). ANOVA with BH correction, significance level p ≤ 0.05. B) Upregulation of PDGFRA gene expression in IDH1-mut versus IDH1-wt AML patients in RNA microarray data (GSE6891) [16]. Differential expression analysis of n = 33 IDH1-mut versus n = 387 IDH1-wt AML patients from Affymetrix HG-U133 plus 2 Microarray. Blue = 606 downregulated genes in IDH1-mut vs. IDH1-wt AML with cut-off of p.adjusted < 0.05, red = 837 upregulated genes in IDH1-mut vs. IDH1-wt AML with cut-off of p.adjusted < 0.05. Expression analysis performed using limma package in R version 4.0.3.*

Supplementary Figure S2: Overall survival related to *IDH1/2* mutation status and *PDGFRA* expression (Bamopoulos 2020 and BeatAML dataset)

A **Overall survival of patients with *IDH2*-wt/mut AML and high/low *PDGFRA* expression (Bamopoulos 2020)**

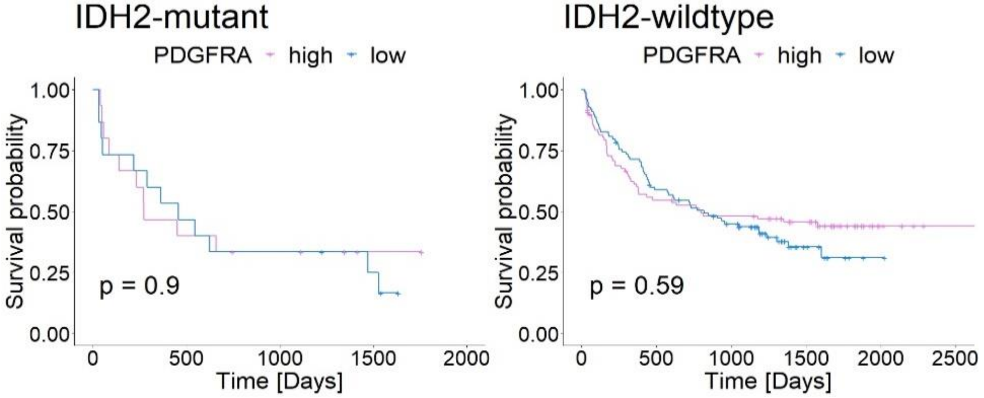

B **Overall survival of patients with *IDH1*-wt/mut or *IDH2*-wt/mut AML and high/low *PDGFRA* expression (BeatAML data)**

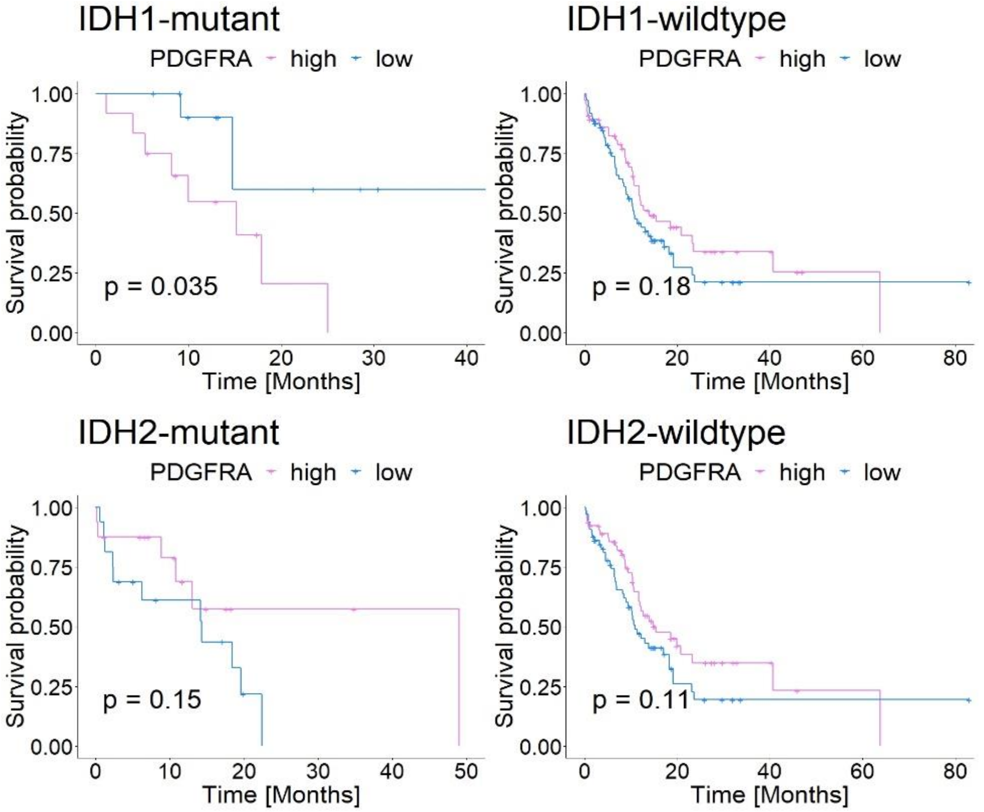

Supplementary Figure S2*: A) Overall survival of IDH2-mut AML patients with high/low PDGFRA expression (n = 15 vs. n = 15) versus IDH2-wt patients with high/low PDGFRA (n = 68 vs. n = 68). PDGFRA high/low: samples with PDGFRA expression above/below median. Method: Kaplan-Maier, significance cut-off p < 0.05. Data: Bamopoulos 2020. B) Overall survival of IDH1-mut patients (PDGFRA high/low: n = 12 vs. n = 12) versus IDH1-wt patients (PDGFRA high/low: n = 65 vs. n = 65) and IDH2-mut patients (PDGFRA high/low: n = 16 vs. n = 16) versus IDH2-wt patients (PDGFRA high/low: n = 65 vs. n = 65). Method: Kaplan-Maier, significance cut-off p < 0.05. Data: BeatAML.*

Supplementary Figure S3: Validation of R132H mutation knock-in and 2-HG production in *IDH1*-mut KG1a single cell clones

A **Sanger sequencing of *IDH1*-wt/-mut KG1a single cell clones**

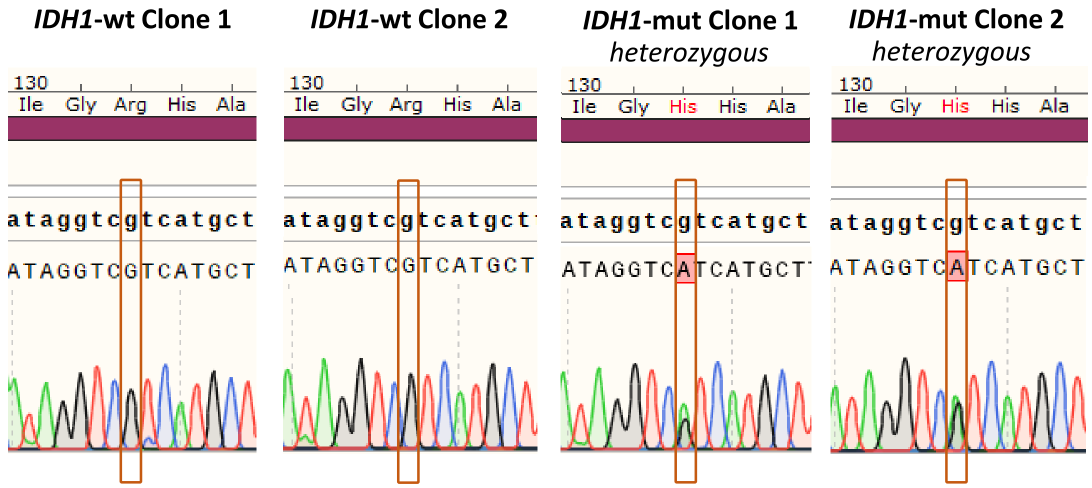

B **2-HG production in *IDH1*-wt/-mut KG1a single cell clones**

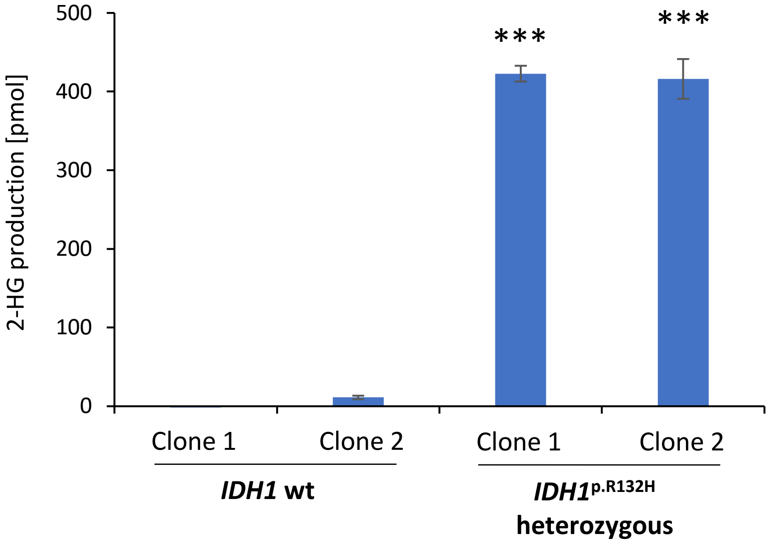

Supplementary Figure S3: *A) Sanger sequencing confirmation of heterozygous IDh1 p.R132H mutation knock-in in KG1a single cell clones. B) 2-HG production quantification of IDH1-mut versus IDH1-wt KG1a single cell clones using fluorescent 2-HG detection assay. 2 clones, 3 replicates, Students T-test * p < 0.05, ** p < 0.01, *** p < 0.001.*

Supplementary Figure S4: Sensitivity of *IDH1*-mut KG1a cells to venetoclax upon ABCB1/MDR1/P-GP efflux pump blockade

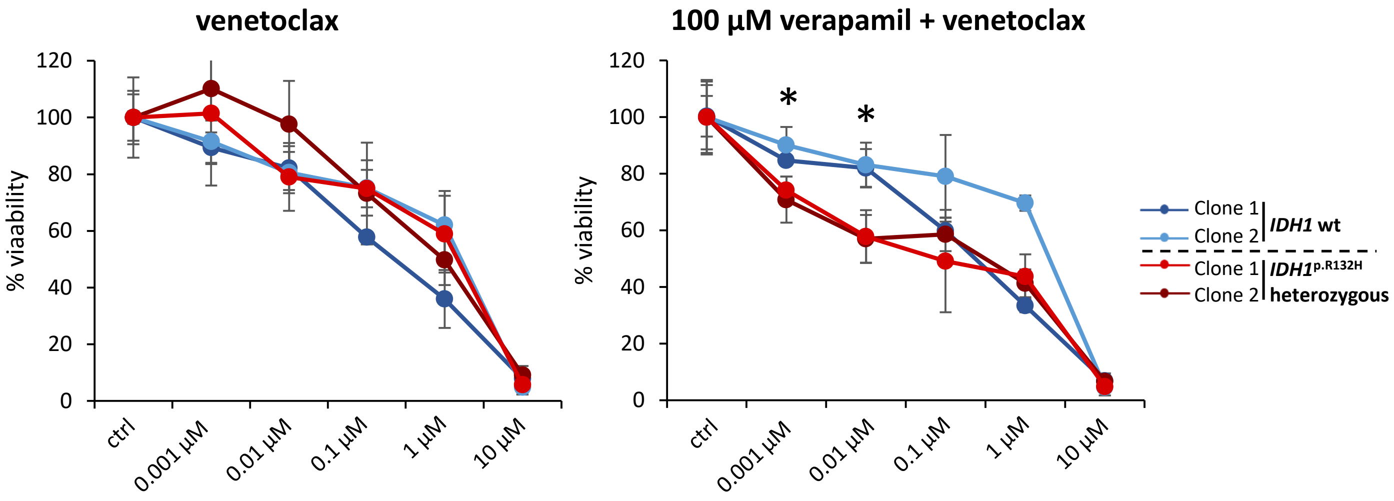

Supplementary Figure S4: *Normalized cell viability (%) of IDH1-wt and IDH1 mut KG1a clones upon venetoclax treatment with or without verapamil-induced ABCB1/MDR1/P-GP efflux pump blockade. 2 clones, 3 replicates, Students T-test * $p < 0.05$.*

Supplementary Figure S5: *PDGFRA* expression and *PDGFRA*-CTCF methylation in *IDH1*-wt/-mut cell clones upon ivosidenib treatment

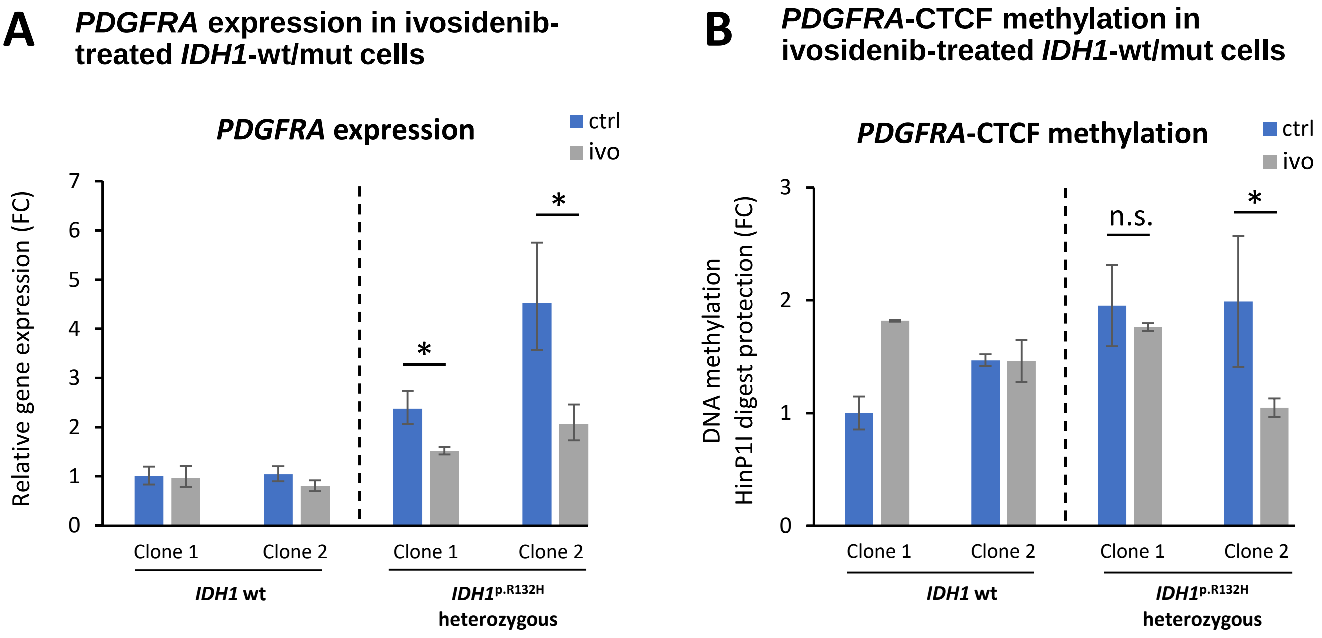

Supplementary Figure S5*: A) RT-qPCR quantification of PDGFRA expression in IDH1-wt/mut clones upon ivosidenib treatment (72h). Data display fold change (FC) in relative expression. 2 clones, 3 replicates, Students T-test * p < 0.05 B) RT-qPCR of PDGFRA-CTCF hypermethylation of IDH1-wt/mut clones upon ivosidenib treatment. Data display fold change (FC) in relative expression. 2 clones, 3 replicates, Students T-test * p < 0.05, ** p < 0.01, *** p < 0.001.*

Supplementary Figure S6: Cell viability, *PDGFRA* expression and *PDGFRA*-CTCF methylation in *IDH1*-wt/-mut cell clones upon 5-azacytidine treatment

A ***Sensitivity of IDH1-wt/-mut cells to*** **5-azacytidine**

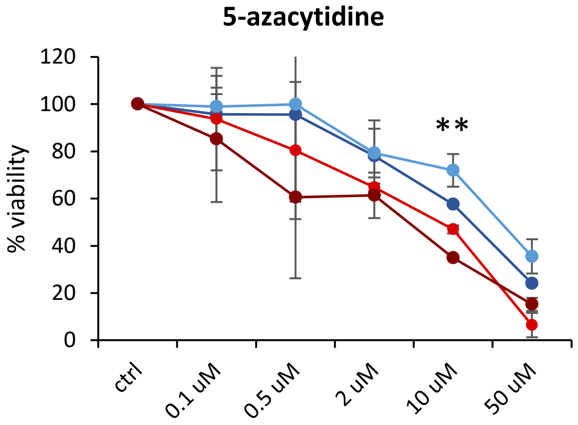

B ***PDGFRA*** **expression in 5-azacytidine-treated *IDH1*-wt/mut cells**

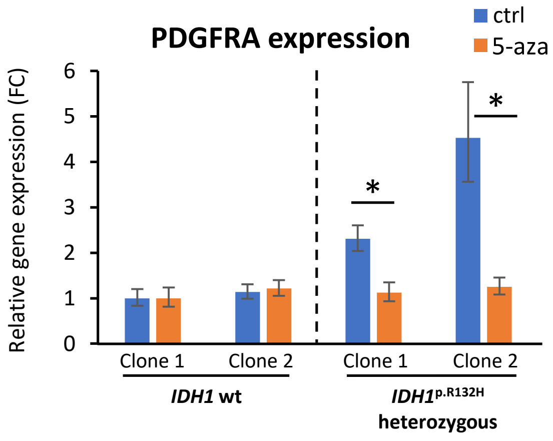

C ***PDGFRA*-CTCF methylation in 5-azacytidine-treated *IDH1*-wt/mut cells**

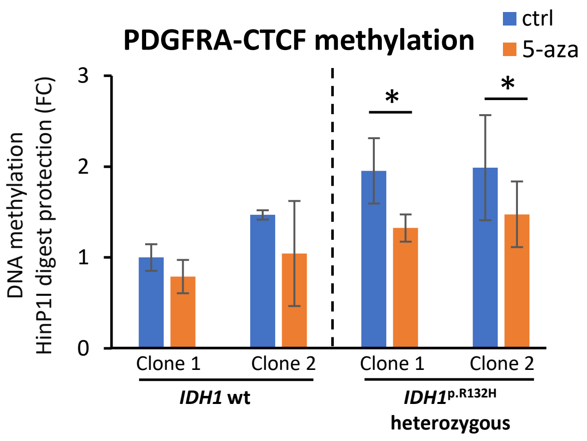

***Supplementary Figure S6**: A) Normalized cell viability (%) of IDH1-wt and IDH1 mut clones upon 72 h treatment with 5-azacytidine. 2 clones, 3 replicates, Students T-test * p < 0.05, ** p < 0.01. B) RT-qPCR quantification of PDGFRA expression in IDH1-wt/mut clones upon 5-azacytidine (72h). Data display fold change (FC) in relative expression. 2 clones, 3 replicates, Students T-test * p < 0.05. C) RT-qPCR of PDGFRA-CTCF hypermethylation of IDH1-wt/mut clones upon 5-azacytidine. Data display fold change (FC) in relative expression. 2 clones, 3 replicates, Students T-test * p < 0.05.*

Supplementary Figure S7: *PDGFRA* expression in *IDH1*-mut AML cells after ivosidenib treatment

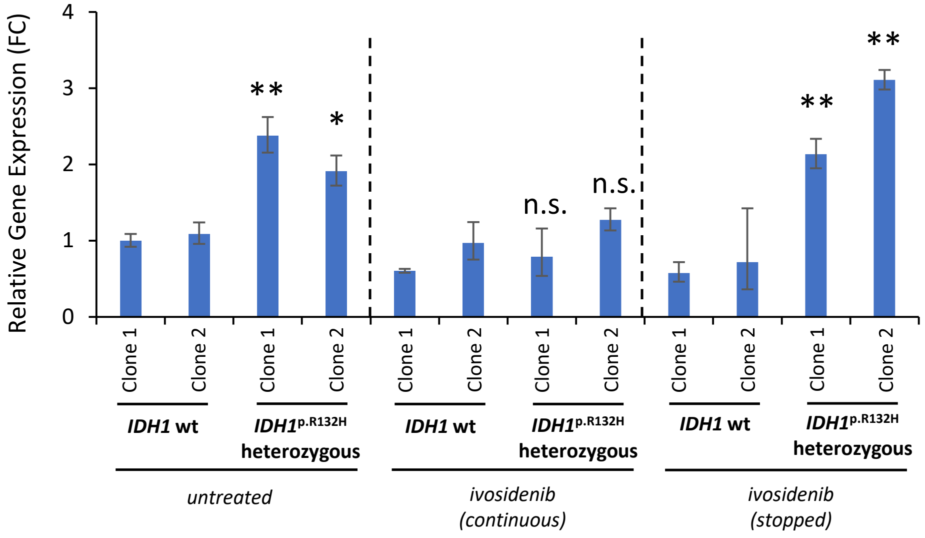

Supplementary Figure S7*: RT-qPCR quantification of PDGFRA expression in IDH1-mut AML cells after 36h ivosidenib treatment versus 36h after treatment pause. Data display fold change (FC) in relative expression. 2 clones, 3 replicates, Students T-test * p < 0.05, ** p < 0.01.*

Supplementary Figure S8: *IDH1*-mut KG1a cell viability upon ivosidenib and dasatinib treatment using FACS-based cell counting

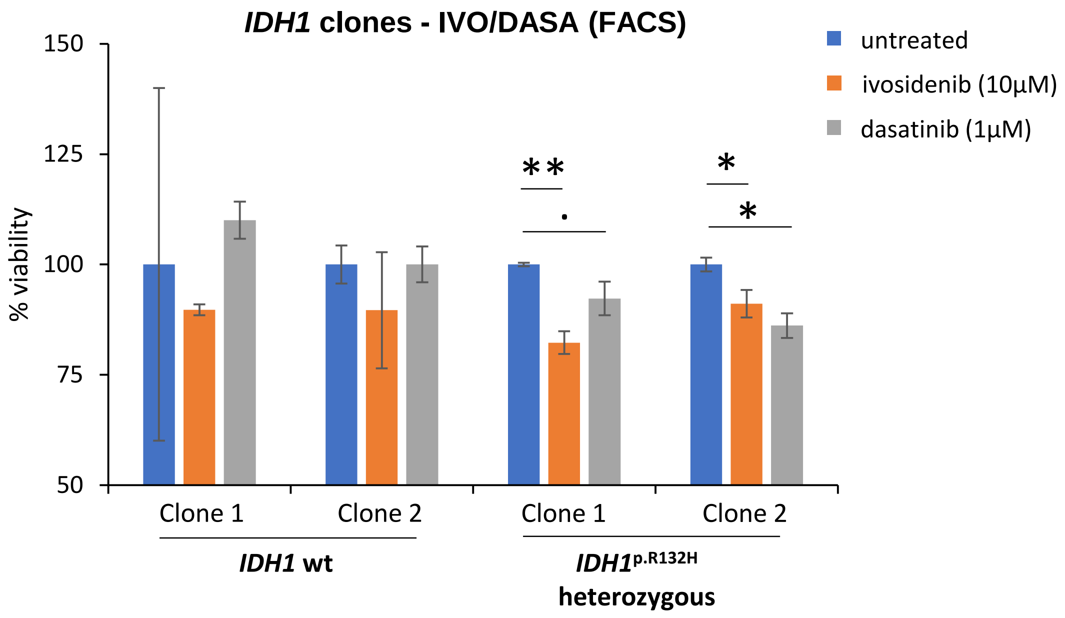

Supplementary Figure S8: *Normalized cell viability (%) of IDH1-wt and IDH1 mut clones upon 72 h treatment with ivosidenib and dasatinib, based on FACS-based counting of viable cells. 2 clones, 3 replicates, Students T-test * p < 0.05, ** p < 0.01.*

Supplementary Figure S9: Confirmation of CRISPR knockout of *PDGFRA in IDH1*-mut KG1a cells and *PDGFRA*-CTCF knockout in *IDH1*-wt KG1a cells and sensitivity of IDH1-wt/-mut KG1a cells to TKI crenolanib

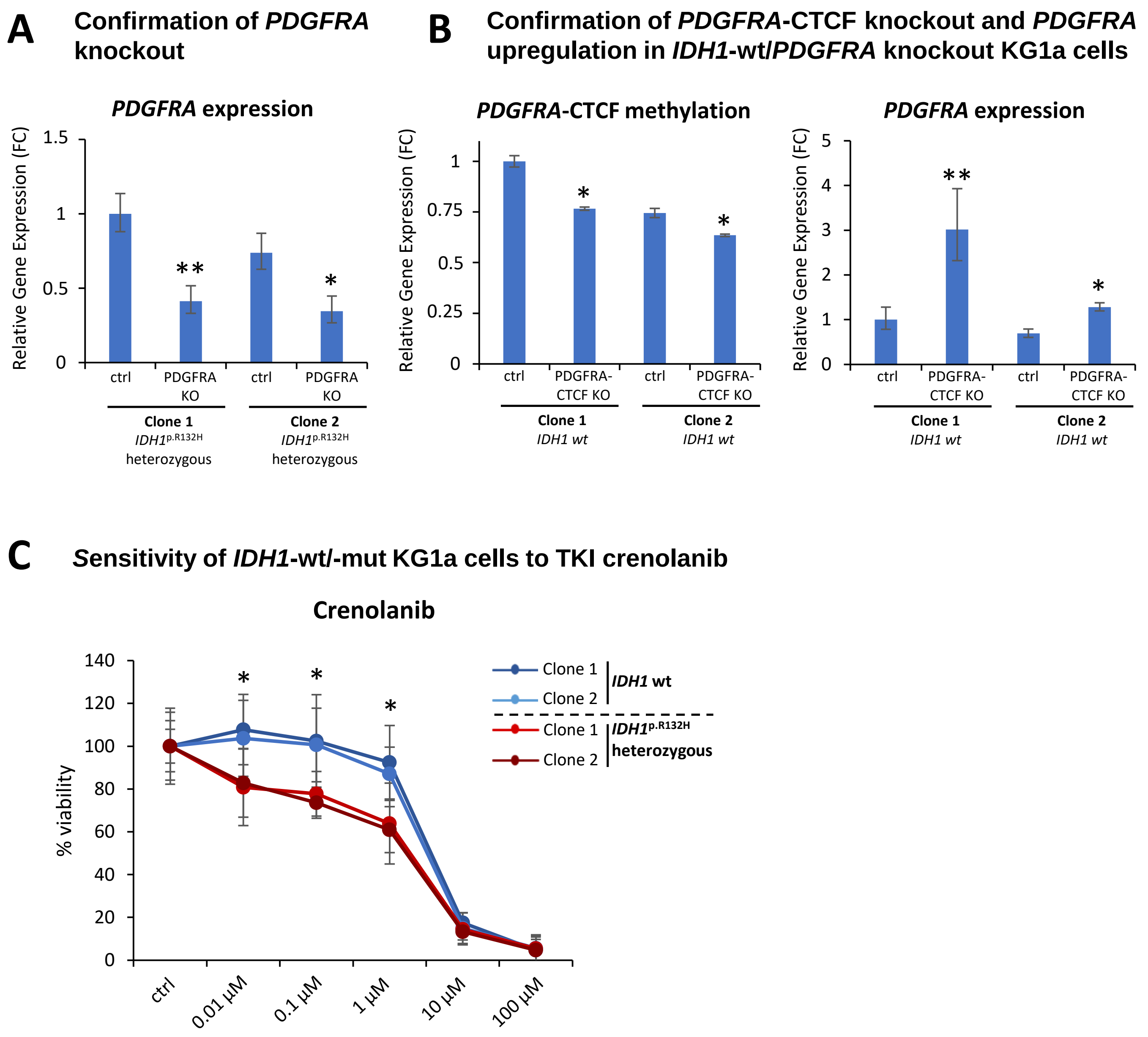

Supplementary Figure S9*: A) RT-qPCR confirmation of PDGFRA knockout in IDH1-wt KG1a cells. Data display fold change (FC) in relative expression. 2 clones, 3 replicates, Students T-test * p < 0.05, ** p < 0.01. B) RT-qPCR confirmation of PDGFRA-CTCF knockout and subsequently upregulated PDGFRA expression in IDH1-mut cells. Data display fold change (FC) in relative expression. 2 clones, 3 replicates, Students T-test * p < 0.05, ** p < 0.01.* C) *Normalized cell viability (%) of IDH1-wt and IDH1-mut KG1a clones upon treatment with TKI crenolanib (72 h). 2 clones, 3 replicates, Students T-test * p < 0.05.*

Supplementary Figure S10: Drug screening *IDH1*-wt/-mut clones for ivosidenib, dasatinib and 5-azacytidine as mono and combinational treatment

A Combinational treatment of *IDH1*-wt/-mut KG1a cells with ivosidenib + dasatinib

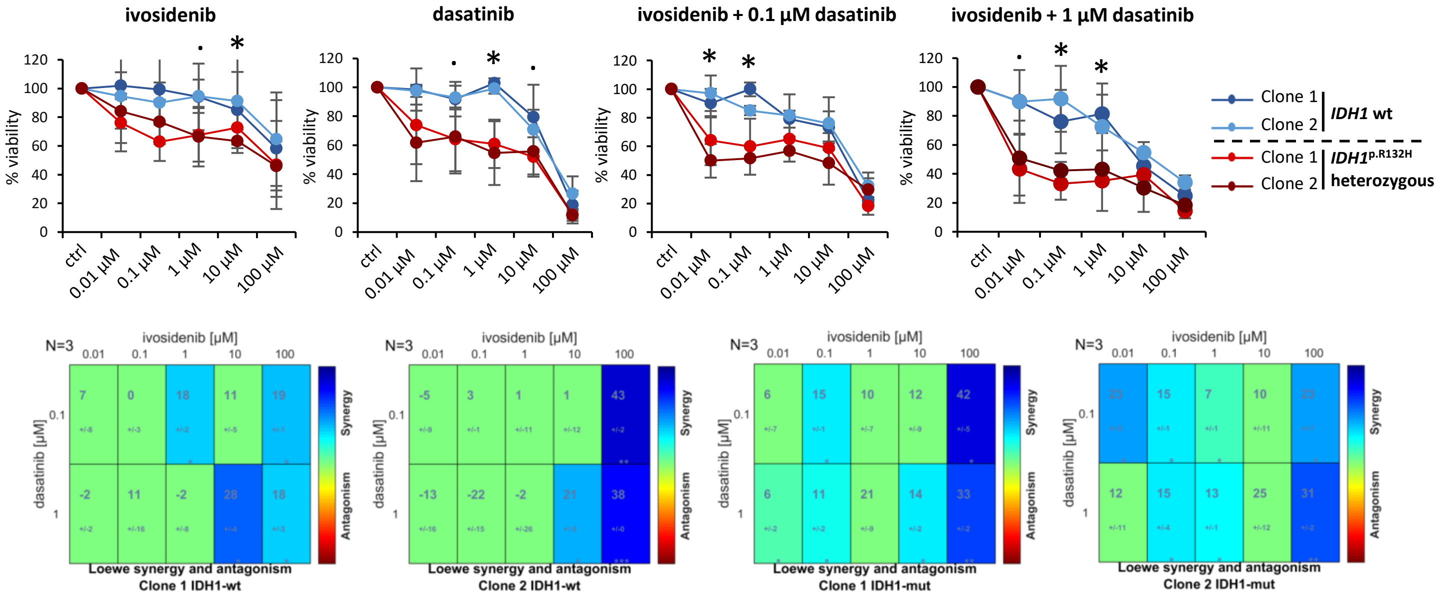

B Combinational treatment of *IDH1*-wt/-mut KG1a cells with ivosidenib + 5-azacytidine

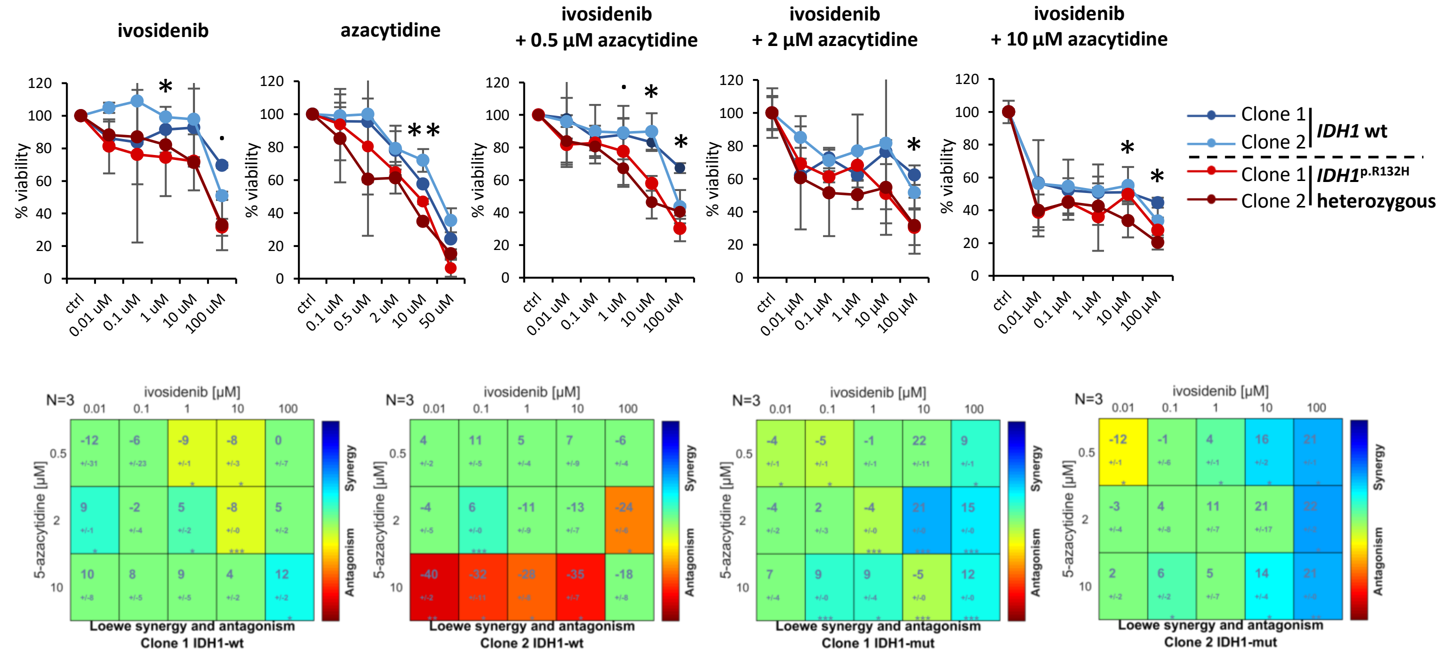

***Supplementary Figure S10**: A) Normalized cell viability (%) of IDH1-wt/mut KG1a cells upon 72 h mono- or combinational treatment with ivosidenib and dasatinib. 2 clones, 3 replicates, Students T-test * p < 0.05, ** p < 0.01. Synergy calculation per clone using Combenefit analysis tool based LOEWE statistical model. B) Normalized cell viability (%) of IDH1-wt/mut KG1a cells upon 72 h mono- or combinational treatment with ivosidenib and 5-azacytidine, 2 clones, 3 replicates, Students T-test * p < 0.05, ** p < 0.01. Synergy calculation per clone using Combenefit analysis tool based LOEWE statistical model.*

Supplementary Figure S11: *S*ensitivity of *IDH1*-mut KG1a cells to sequential treatment with 5-azacytidine/ivosidenib followed by dasatinib

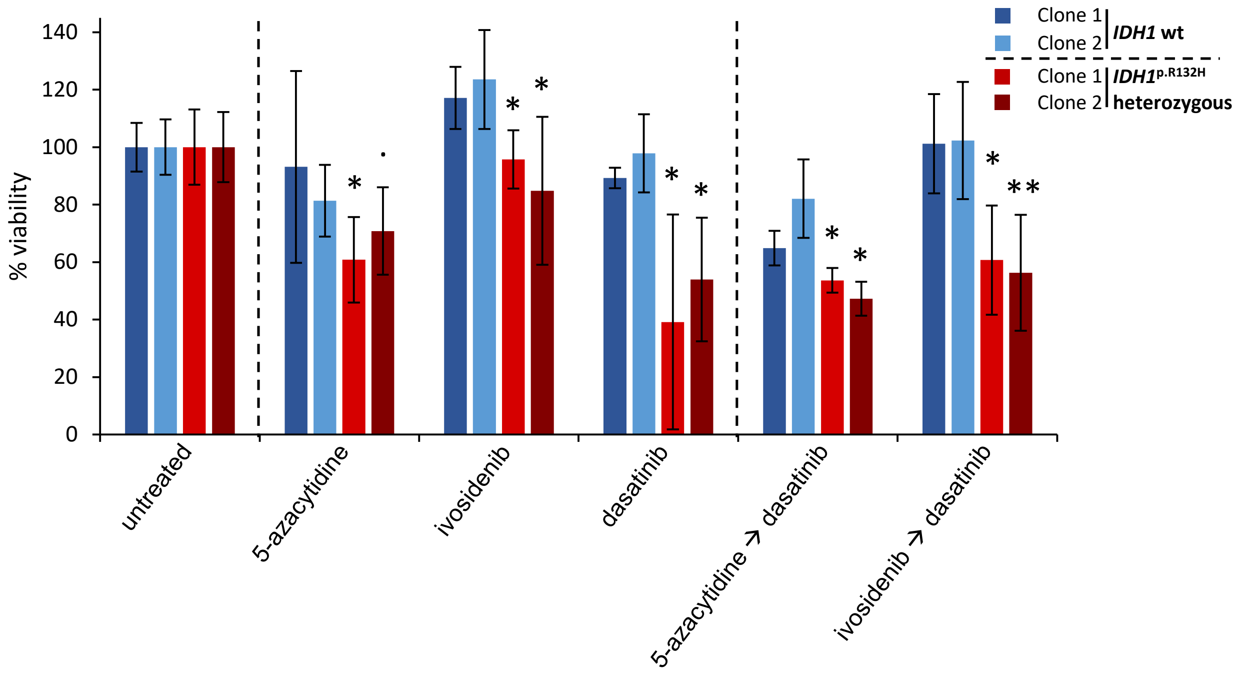

Supplementary Figure S11*: Normalized cell viability (%) of IDH1-wt/mut KG1a cells upon 72 h mono-treatment with 5 µM 5-azacytidine, 5 µM ivosidenib and 1 µM dasatinib versus sequential treatment (36h/36h) with 5-azacytidine → dasatinib or ivosidenib → dasatinib. 2 clones, 3 replicates, Students T-test * $p < 0.05$, ** $p < 0.01$.*
